# Assessment of optogenetically-driven strategies for prosthetic restoration of cortical vision in large-scale neural simulation of V1

**DOI:** 10.1038/s41598-021-88960-8

**Published:** 2021-05-24

**Authors:** Jan Antolik, Quentin Sabatier, Charlie Galle, Yves Frégnac, Ryad Benosman

**Affiliations:** 1 Faculty of Mathematics and Physics, Charles University, Malostranské nám. 25, 118 00 Prague 1, Czechia; 2 Sorbonne Université, INSERM, CNRS, Institut de la Vision, 17 rue Moreau, 75012 Paris, France; 3 Unité de Neurosciences, Information et Complexité (UNIC), NeuroPSI, Gif-sur-Yvette, France; 4 University of Pittsburgh, McGowan Institute, 3025 E Carson St, Pittsburgh, PA USA

**Keywords:** Network models, Computer science

## Abstract

The neural encoding of visual features in primary visual cortex (V1) is well understood, with strong correlates to low-level perception, making V1 a strong candidate for vision restoration through neuroprosthetics. However, the functional relevance of neural dynamics evoked through external stimulation directly imposed at the cortical level is poorly understood. Furthermore, protocols for designing cortical stimulation patterns that would induce a naturalistic perception of the encoded stimuli have not yet been established. Here, we demonstrate a proof of concept by solving these issues through a computational model, combining (1) a large-scale spiking neural network model of cat V1 and (2) a virtual prosthetic system transcoding the visual input into tailored light-stimulation patterns which drive in situ the optogenetically modified cortical tissue. Using such virtual experiments, we design a protocol for translating simple Fourier contrasted stimuli (gratings) into activation patterns of the optogenetic matrix stimulator. We then quantify the relationship between spatial configuration of the imposed light pattern and the induced cortical activity. Our simulations in the absence of visual drive (simulated blindness) show that optogenetic stimulation with a spatial resolution as low as 100 $\upmu$m, and light intensity as weak as $10^{16}$ photons/s/cm$^2$ is sufficient to evoke activity patterns in V1 close to those evoked by normal vision.

## Introduction

Several significant efforts have been undertaken to develop prosthetic implants for restoring vision in blind patients^1–7^. Devices that target the retina^8–12^ circumvent disorders at photo receptor levels such as macular degeneration or retinitis pigmentosa. However, vision restoration in conditions like glaucoma, diabetic retinopathy or trauma, cannot be achieved through prosthetic intervention at the retina level^5^. In these cases, targeting processing stages further down the visual stream, notably lateral geniculate nucleus (LGN) or V1, becomes necessary^1,2,4,7^.

Numerous cortical stimulation technologies are available or under development^3^. Here we will focus on the case of optogenetic stimulation^13^ that may avoid some of the challenges of direct electrical stimulation by providing more selective neuronal activation, and prevent tissue morbidity associated with long-term application of electrical currents and inflammatory implant-induced injury^11,12,14,15^.

**Figure 1:**
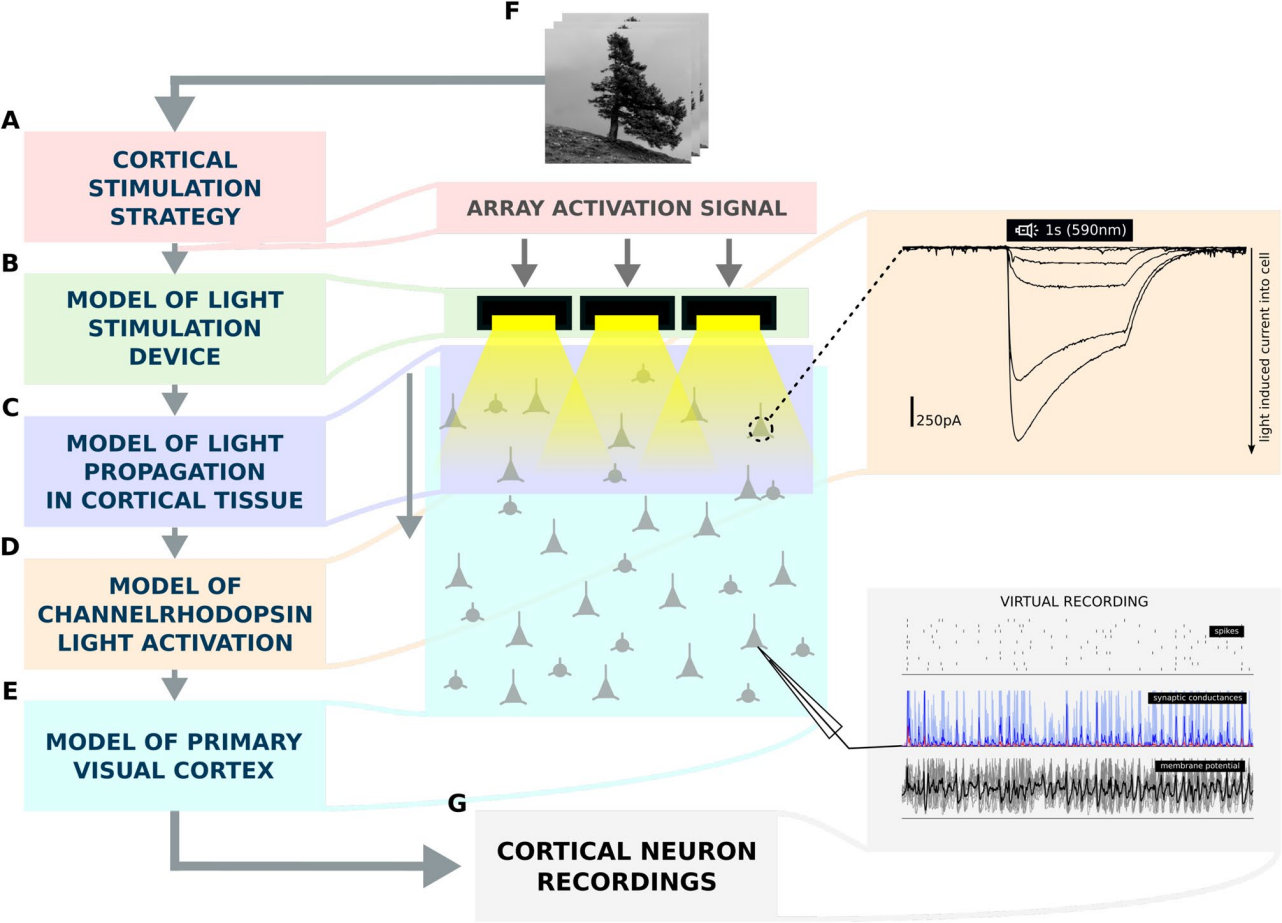
The schematic of the virtual cortical prosthesis experiment. (**A**) The stimulation strategy that translates a class of visual stimuli into driving signals for a MLEE, (**B**) a model of the MLEE, (**C**) a model of light propagation through cortical tissue taking into consideration the absorption and diffraction of the light in neural substrate. (**D**) A model of channelrhodopsin (ChR) dynamics in transfected cells that transforms a temporal trace of light impinging onto a given cell into a current that is injected into the cell due to the activation of ChR channels, and (**E**) a detailed large-scale spiking conductance based neural model of 5 mm$^2$ of primary visual cortex. These 5 components, allow us to simulate the activation of the cortical population (**G**) to a specific set of visual stimuli (**F**). The system allows us to compare the cortical activation patterns elicited by the direct intra-cortical optogenetic stimulation protocol to visually-driven patterns during normal vision.

In the context of sight restoration using an optogenetic stimulation strategy, a region of the cortex would be transfected with channelrhodopsin (ChR), to make the cells excitable by light^16,17^. Patterns of light generated by a matrix of light emitting elements (MLEE)^18–20^ placed on the surface of the treated cortical region (see Fig. 1) would elicit an analogous pattern of spiking activity in the cortex under the implant. The underlying assumption is that, if the imposed cortical spiking activity is similar to the encoding of the visual stimulus under normal vision, a naturalistic perception of that stimulus would be induced at the behavioural/cognitive level.

The implementation of such a hypothetical optogenetics based prosthetic system faces numerous technological challenges. Two-photon optogenetic stimulation is currently the only method with single cell resolution^21–23^. Combined with holographic wavefrontshaping techniques to generate distributed light patterns^24–28^, it allows manipulation of neural circuits in 3D^29,30^. A potential drawback is that effective stimulation of neurons is limited to supra-granular layers^23^, due to the scatter and absorption of light as it propagates through neural tissue^31^. Furthermore, in humans, most of the central visual field representation in V1 is located deep inside the calcarine fissure^32^, out of reach from superficial implants positioned under the skull or interhemispherically. Finally, to effectively engage the visual encoding of neurons under the implant, one needs to have a prior knowledge of their natural stimulus preference. In the blind subject, this knowledge can be acquired through behavioural feedback of subjects to external stimulation; such an approach is, however, time-consuming, imprecise^1,2^ and might not scale to higher-resolution devices.

Nevertheless, despite steady progress in addressing many of these challenges (see Discussion), one of the fundamental questions has received surprisingly little attention: how can one engineer a high-resolution BCI stimulation paradigm to generate a cortical-like encoding directly in V1 similar to that evoked by elementary visual stimuli during normal vision. Such interfacing protocols are challenging to develop due to the limitations of the prosthetic system itself, the complexity of visual stimulus encoding schemes in V1, and the lack of a multi-scale biophysical understanding of the interactions of externally induced optogenetically-driven activation with the ongoing inherent recurrent dynamics within the cortex. Nevertheless, solving this issue is urgent, as simplified versions of such stimulation protocols will be needed for any potential future validation experiments in animal models.

The aim of our study is to provide a detailed proof of concept model using a simulation platform to explore the potential neural effects of cortical prosthetic stimulation protocols in higher mammals. Specifically, we have created a detailed simulation of cortical dynamics in a virtual population of transfected V1 neurons and study its dependence on light stimulation parameters by combining models of the light propagation in cortical tissue, the ChR dynamics model, and a large-scale model of anatomically and functionally calibrated cat V1 cortical circuitry (Fig. 1). Using this simulation, we propose an optogenetic stimulation strategy for reproducing the spike-based encoding of sinusoidal grating visual stimuli in V1. We then perform a systematic characterization of the optogenetically evoked neural dynamics and their similarity to analogous activity patterns evoked by this canonical low dimensional input received from the retina.

To the best of our knowledge, this study represents the first quantitative examination of optogenetic stimulation in a model of visual cortical circuitry. It offers predictions that can guide future experiments, and provides a ready-to-use test bed for potential future in-vivo animal experiments of optogenetic prosthetic systems. Our simulation platform can be easily reconfigured for parameters of human visual system opening the possibility for optimizing the stimulation design for future clinical trials.

## Results

**Figure 2:**
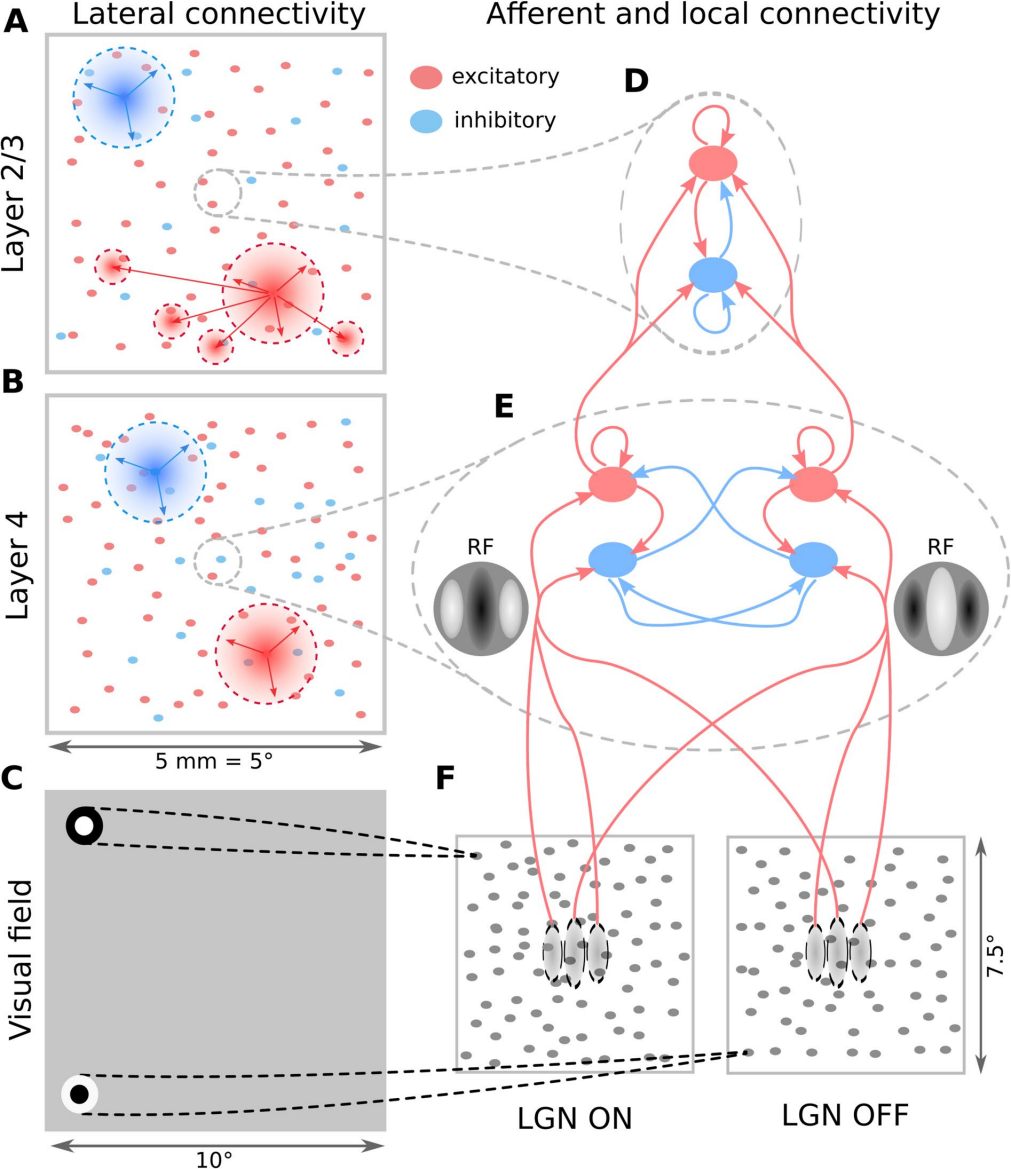
The V1 model architecture. (**A** and **B**) Layer 2/3 and Layer 4 lateral connectivity. All cortical neurons make local connections within their layer. Layer 2/3 excitatory neurons also make long-range functionally specific connections. For the sake of clarity A and B do not show the functional specificity of local connections and connection ranges are not to scale. (**C**) Extent of the modelled visual field and example of RFs of one ON and one OFF-center LGN relay neuron. As indicated, the model is retinotopically organized. (**D**) Local connectivity scheme in Layer 2/3: connections are orientation- but not phase-specific, leading to predominantly Complex cell type RFs. Both neuron types receive narrow connections from Layer 4 excitatory neurons. (**E**) Local connectivity in Layer 4 follows a push-pull organization. (**F**) Afferent RFs of Layer 4 neurons are formed by sampling synapses from a probability distribution defined by a Gabor function overlaid on the ON and OFF LGN sheets, where positive parts of the Gabor function are overlaid on ON and negative on OFF-center sheets. The ON regions of RFs are shown in white, OFF regions in black.

We have developed a simulation environment (Fig. 1) comprising of a model of early visual system (Fig. 2; see Methods "The large-scale spiking model of primary visual cortex" section) combined with a model of a virtual optogenetic-based prosthetic device (see Methods "The model of light emitting elements and light propagation in cortical tissue" and "The channelrhodopsin model" sections). In this study, we propose a light stimulation protocol designed to evoke spiking activity patterns in optogenetically treated V1 to mimic those elicited by drifting sinusoidal gratings during normal vision. To assess the protocol, we will compare simulated cortical activity elicited by a stimulus presented via retina (natural vision) with 3 conditions of simulated cortical activity evoked by the optogenetic stimulation protocol (prosthetic vision). Specifically, we will consider following four conditions:

1. Visually-driven stimulation via retino-thalamic pathway simulating natural vision (NatVis).
2. Optogenetically-driven stimulation in a model with no connectivity (OptoDis).
3. Optogenetically-driven stimulation with disabled thalamic input where only excitatory cells express ChR (OptoExc).
4. Optogenetically-driven stimulation with disabled thalamic input where both excitatory and inhibitory neurons express ChR (OptoExcInh).

**Figure 3:**
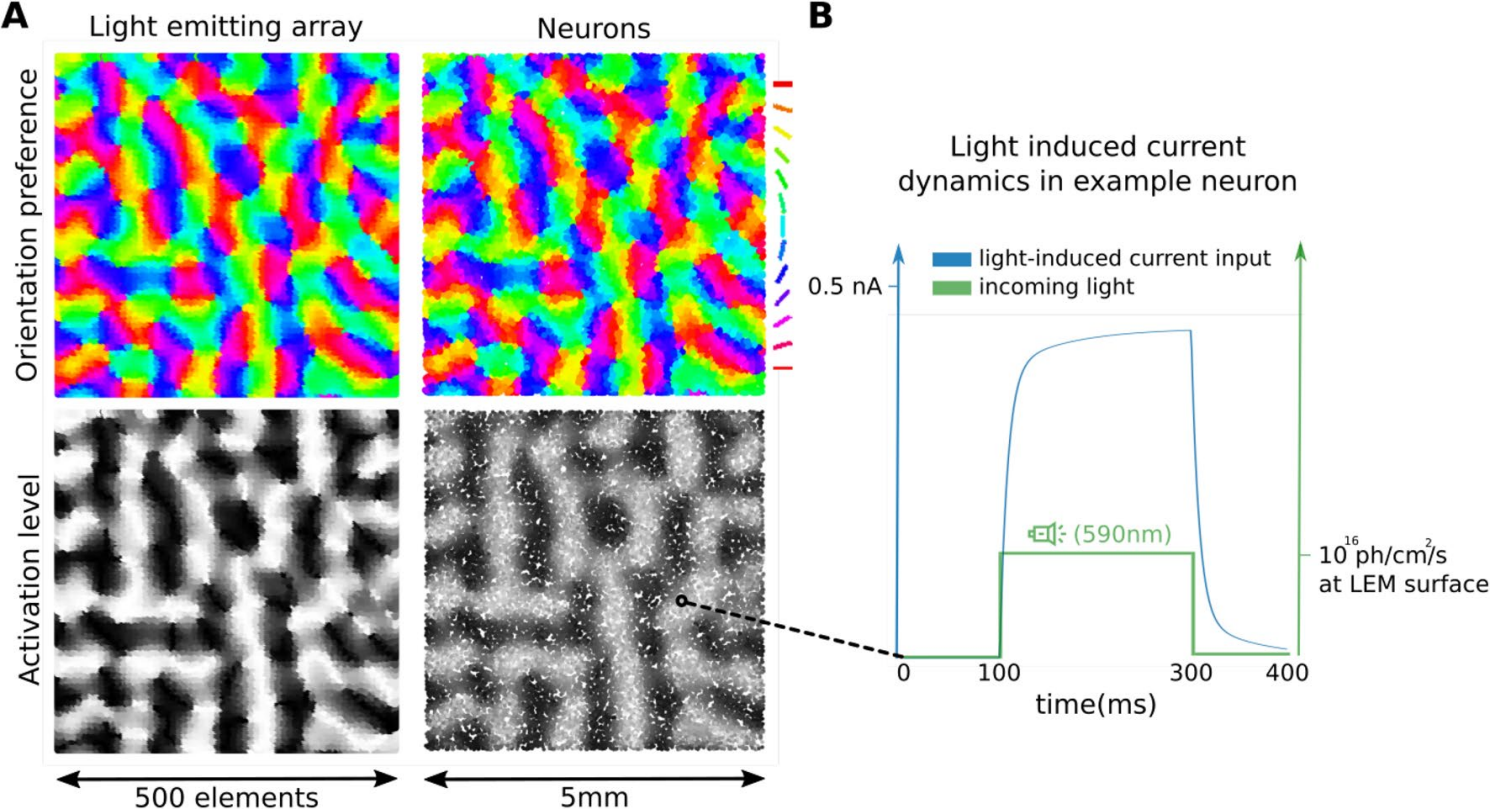
Opto-stimulation protocol for grating stimului. (**A**) The assigned orientation preference to individual light emitting elements and the orientation preference of neurons (top row). The driving signal to individual light-emitting elements imposed by the optogenetic stimulation protocol and the resulting depolarization in cortical tissue (bottom row). (**B**) The light output of an example light-emitting element (green) and the resulting light-mediated inward current to an example neuron located at the same location (blue).

The detailed description of the stimulation protocol can be found in Methods "The stimulation strategy" section, but briefly: we will assume stimulation of layer 2/3 neurons^23^. Layer 2/3 is predominantly composed of complex cells^34,35^ that respond with steady depolarization to stimulation with an optimally oriented drifting sinusoidal grating. Assuming the knowledge of orientation maps under the virtual implant (Fig. 3A), the protocol will illuminate given cortical location with a square optical impulse lasting the duration of the grating stimulus (Fig. 3B,S4AB), and of magnitude inversely proportional to the difference between the orientation of the grating stimulus and the preferred orientation at the given cortical location (Fig. 3A,S4G).

### Time course of the response to visual and optogenetic stimulation

**Figure 4:**
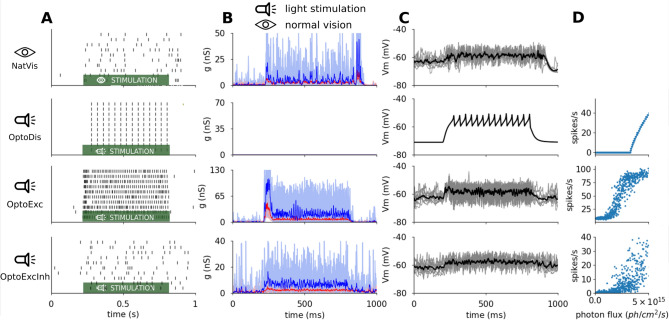
Example single-neuron dynamics in the 4 experimental conditions. The neurons respond to 10 trials of stimulation with a drifting sinusoidal grating or its light-stimulation equivalent, that starts at 200 ms and stops at 800 ms. From top to bottom NatVis, OptoDis, OptoExc, and OptoExcInh conditions. (**A**) Spike raster plot. (**B**) Excitatory (red) and inhibitory (blue) conductances. (**C**) Membrane potential. (**D**) The relationship between photon-flux and the spiking response of neurons in the given condition. In (**B**) and (**C**) pale thin lines are single-trials, thick saturated lines are mean across trials. In all three optogenetic conditions an arbitrary stimulation scaling factor of $L_{max}= 9.2 \times 10^{15}$ photons/s/cm$^2$ was used.

We will first compare neural response in a representative neuron to 600 ms presentation of an optimal sinusoidal grating or its optogenetically driven equivalent across the four conditions (Fig. 4). Neurons in all conditions respond to the stimulation by a tonic depolarization for the duration of the stimulus, but beyond this coarse characteristic, a number of differences are apparent. Different levels of membrane potential depolarization ($V_m$), patterns of variance and ratios of excitatory and inhibitory conductance are noticeable. These will be analysed in section "Statistical properties of the opogenetically evoked cortical activity".

#### Absence of OFF dynamics in optogenetically-driven conditions

Transient ON and OFF dynamics can be observed in the NatVis condition (Fig. 4B top panel) after the on-set and off-set of the stimulus. These observations are in line with experimental evidence^36,37^. But in the three optogenetically-driven conditions only the ON dynamics are present (Fig. 4B bottom two panels). Under normal vision, the temporally bi-phasic nature of ON and OFF LGN cells RFs^38^, included also in our model, contribute to the generation of the ON and OFF dynamics in V1^36,39,40^. These feed-forward mechanisms are, however, not engaged during optogenetic stimulation. It is thus not surprising that, given that the proposed light stimulation protocol is not explicitly designed to induce them, the OFF dynamics are not present in the optogenetic condition. Interestingly, we observe that the optogenetic stimulation (OptoExc, OptoExcInh conditions) does induce qualitatively similar ON dynamics in V1 neurons, as per the different temporal evolution of excitation and inhibition during external stimulation.

#### Differences in sensitivity to light intensity accross the optogenetically-driven conditions

In all three optogenetic conditions (OptoDis, OptoExc, OptoExcInh) the same test light intensity profile was used to stimulate the cortex (Fig. 4). It can be noted, however, that there are major differences in the overall firing response across the 3 conditions (Fig. 4A). In the OptoDis condition, lack of spontaneous noise leads to a steep illumination-spiking curve (Fig. 4D), with a sharp threshold determined by the difference between resting membrane potential and spiking threshold (G=2.62 , eT=3.00 $\times 10^{15}$ photons/s/cm$^2$, see Methods section "Data Analysis"). In the two fully connected optogenetic conditions (OptoExc, OptoExcInh), the ongoing bombardment of spikes from thalamo-cortical and cortico-cortical pathways induces additional variability in $V_m$ of modelled cortical neurons. This means that even small levels of light-induced depolarization will generate extra spikes. Thus, in the OptoExc condition the illumination-spiking curve is shifted to the left with smoother threshold transition relative to the OptoDis condition (G=1.82 , eT=0.51 $\times 10^{15}$ photons/s/cm$^2$, see Methods section "Data Analysis"). Furthermore, the increased variability of $V_m$ in the fully connected model implies that the same mean level of depolarization will result in higher response rates in the OptoExc condition. Finally, in the OptoExcInh condition, we can observe lower gain and higher thresholds in the illumination-spiking curve (G=1.02 , eT=1.71 $\times 10^{15}$ photons/s/cm$^2$, see Methods section "Data Analysis"), in comparison to the OptoExc condition, due to the additional inhibitory drive induced by the direct optogenetic stimulation of the inhibitory neurons.

These differences in overall responsiveness to the same levels of light stimulation across the 3 optogenetic conditions pose a challenge in comparing their responses. To address this issue, we have devised a method for mapping the desired level of spiking output in the stimulated cortex to the light level that will induce it (see supplementary methods section 1.2). In the remainder of the study, we use this protocol to adjust the overall level of light stimulation individually for each optogenetic condition, such that the average response levels in each optogenetic condition approximately match those in the NatVis condition.

### Optogenetically induced orientation tuning of cortical responses

#### NatVis condition

**Figure 5:**
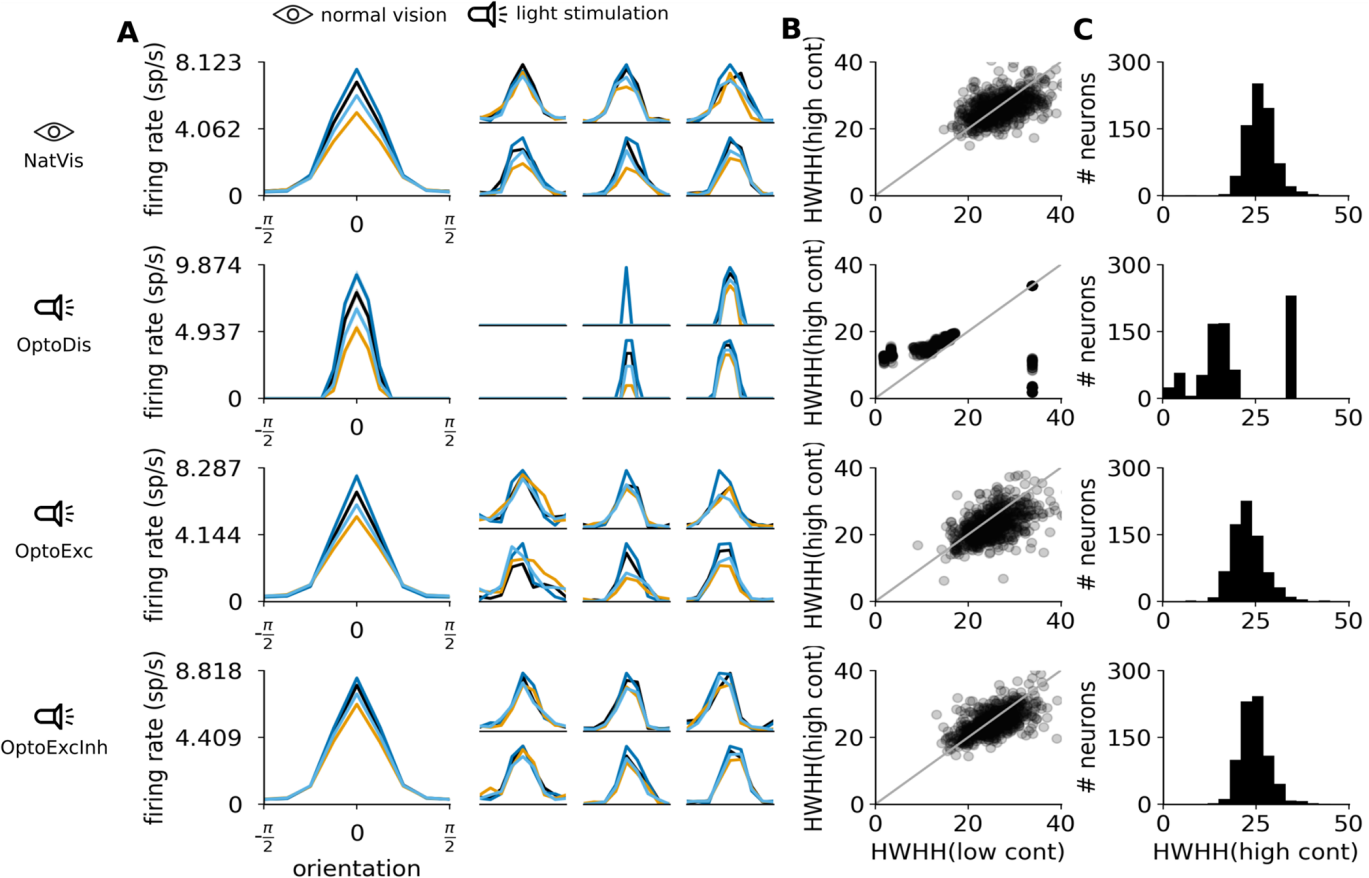
Orientation tuning of model neurons in the 4 experimental conditions. From top to bottom NatVis, OptoDis, OptoExc, and OptoExcInh conditions. (**A**) Mean orientation tuning curves across all recorded neurons (larger plot) and examples of individual tuning curves in 6 randomly selected neurons (smaller plots). (**B**) Scatter plot of half-height at half-width (HWHH) of orientation tuning curves at low (abscissa) versus high (ordinate) contrast. (**C**) Distribution of HWHH at high contrast intensity.

The cortical circuit model^33^ upon which the present study is based has been shown to match the main features of orientation tuning properties of V1 neurons, including the response level at orthogonal orientation^41^ close to spontaneous activity level, or contrast invariance of tuning width^41–43^ (figure S2). Indeed, orientation selectivity measurements in the NatVis condition show that excitatory neurons in model layer 2/3 express Gaussian shaped (Fig. 5A top panel) contrast invariant (Fig. 5B top panel) orientation tuning with a mean half-width at half-height (HWHH) of about 25 degrees (Fig. 5C), in agreement with the experimental literature.

#### OptoDis condition

The responses in the OptoDis condition are orientation tuned, but their width increases with stimulus contrast (Fig. 5). Furthermore, increasing the light intensity beyond the levels dictated by the contrast calibration (see supplementary methods section 1.2) would further broaden the tuning curves, eventually eliciting significant responses at orthogonal orientations (figure S6C,D). This is because in cortical tissue light disperses into nearby domains that are not meant to be illuminated. On the other hand, due to the lack of spontaneous activity in the decoupled model, the gap between the resting membrane potential $V_m$ and spiking threshold causes an iceberg effect, whereby illumination below the fixed threshold generates zero response. This leads to very narrow tuning curves that abruptly cross the zero response level at low overall illumination levels. In principle, for fixed contrast, it would be possible to tune the stimulation protocol (parameter $\sigma$, see "The stimulation strategy" section) such that tuning similar to that of NatVis condition is achieved in the OptoDis condition. However, this ad-hoc adjustment can not be done in a contrast independent manner, making it impossible to develop a general purpose stimulation protocol for encoding of arbitrary visual stimuli.

#### OptoExc condition

From Fig. 5 (third row), it can be observed that when the same stimulation protocol is used in the OptoExc condition, the cortical network generates tuning curves with similar tuning width and contrast invariance to those in NatVis condition (Fig. 5, top row). This shows that the recruitment of intra-cortical connectivity, which is known to exert competitive influences—where stronger responses are magnified, while weaker ones suppressed—can compensate for the limited targeting precision of the optogenetic stimulation that can cause unintended stimulation of non-preferred orientation columns^44^. It should however be noted that if the illumination intensity is increased beyond levels dictated by contrast mapping, we do observe non-zero responses at orthogonal orientations and a break-down of the contrast invariance of tuning in the Optogenetic Excitatory Only condition (figure S6A,B).

#### OptoExcInh condition

Next, we have repeated the same optogenetic stimulation protocol but targeting both the excitatory and inhibitory neural populations (OptoExcInh condition). Surprisingly, we find nearly identical outcomes to OptoExc condition, with orientation tuning in this condition exhibiting very similar width and contrast invariance. This indicates that from the point of view of stimulus selectivity, targeting exclusively excitatory neurons does not pose an advantage over targeting both excitatory and inhibitory populations simultaneously.

#### Sharpness of optogentically evoked orientation tuning

**Figure 6:**
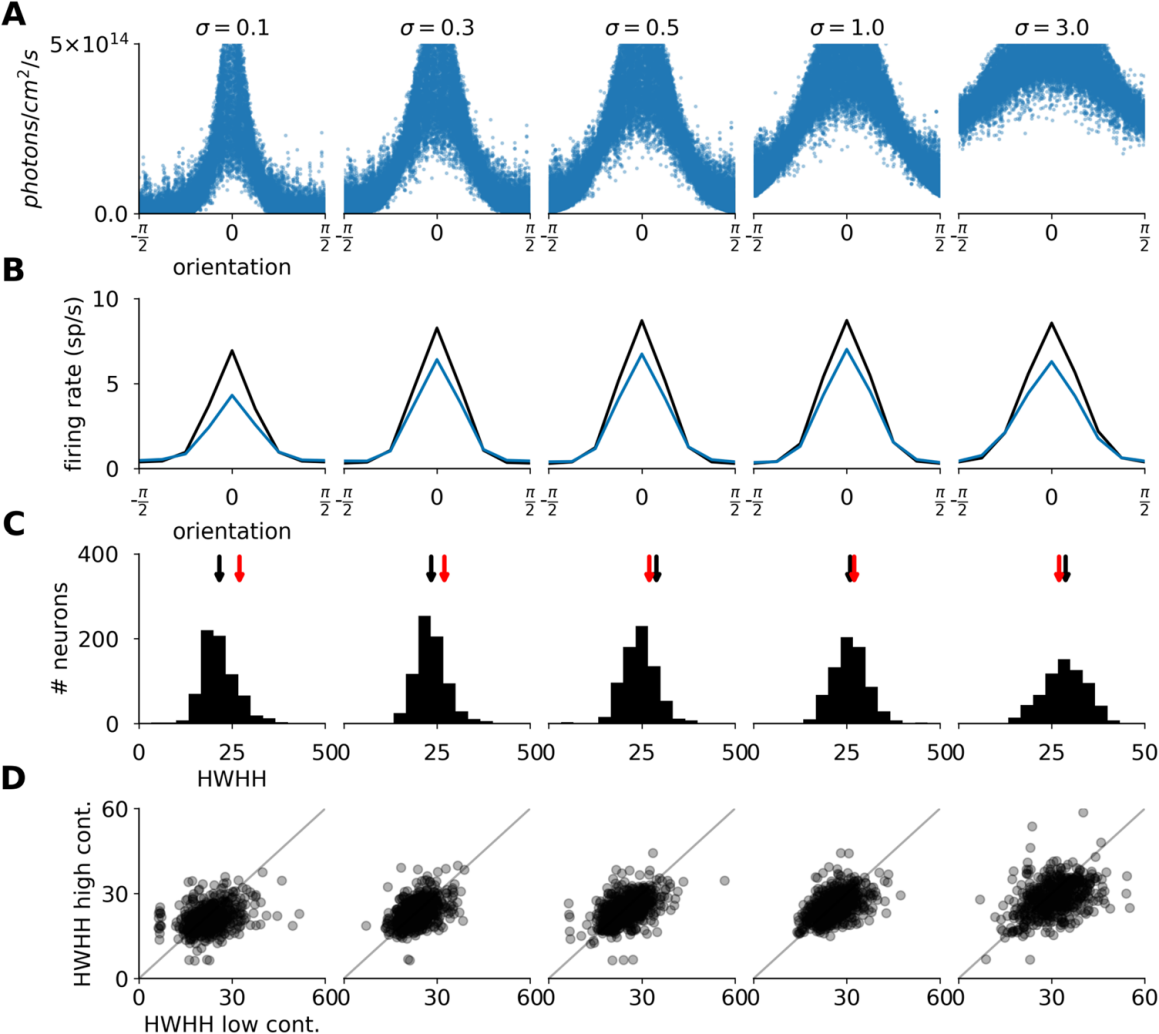
Width of orientation tuning as a function of the sharpness parameter of the optogenetic stimulation protocol in the OptoExc condition. Each column corresponds to cortical optogenetic stimulation simulation where different parameters of the stimulation orientation sharpness $\sigma$ were used. (**A**) Illumination intensity at the cell body as a function of the orientation preference of the given neuron. (**B**) Mean of the orientation tuning curves across all recorded neurons (after realignment on their respective orientation preference). (**C**) The histogram of tuning width measured as HWHH at maximum contrast. The black arrows on top mark the mean of the distribution. The red arrows mark the mean HWHH of the natural vision condition. (**D**) The scatter plot showing the orientation tuning width measured as HWHH at minimum and maximum contrast.

In the virtual experiments presented so far, we have fixed the sharpness parameter $\sigma$ of the stimulation protocol at an arbitrary value of 0.5 (see methods "The stimulation strategy" section). However, it might be necessary to tune the orientation selectivity of the protocol in a more versatile way, so as to account for the structural differences across species or adapt to the non-linearity of the retino-cortical magnification factor with visual eccentricity. We have examined this issue in the OptoExc condition and found that the width of orientation tuning can be sharpened or broadened in a continuous way by systematically changing the $\sigma$ parameter (Fig. 6B,D).

However, in spite of the broad range of explored $\sigma$ values, the range of resulting orientation tuning width of cortical responses remains narrow. This observation again strengthens the hypothesis that the recurrent intra-cortical processing tends to push the resulting activation patterns towards those observed during normal visual stimulation. Our simulations therefore suggest that the orientation tuning imposed by optogenetically-driven stimulation can be tailored for specific circumstances. Similar behaviour was also observed in the OptoExcInh condition (figure S6).

### Statistical properties of the opogenetically evoked cortical activity

As apparent in Fig. 4, higher-order statistical properties of both sub- and supra-threshold neural signals differ markedly across the conditions. Since higher-order statistics of spiking response could have perceptual implications, a closer investigation is needed. In this section we will compare several statistical measures of sub- and supra-threshold neural signals between the NatVis and the two fully-connected optogenetic (OptoExc, OptoExcInh) conditions (due to the lack of meaningful variability, OptoDis condition does not offer any useful insight, and is therefore omitted from this analysis).

#### Reduced synchronization in optogenetic conditions

**Figure 7:**
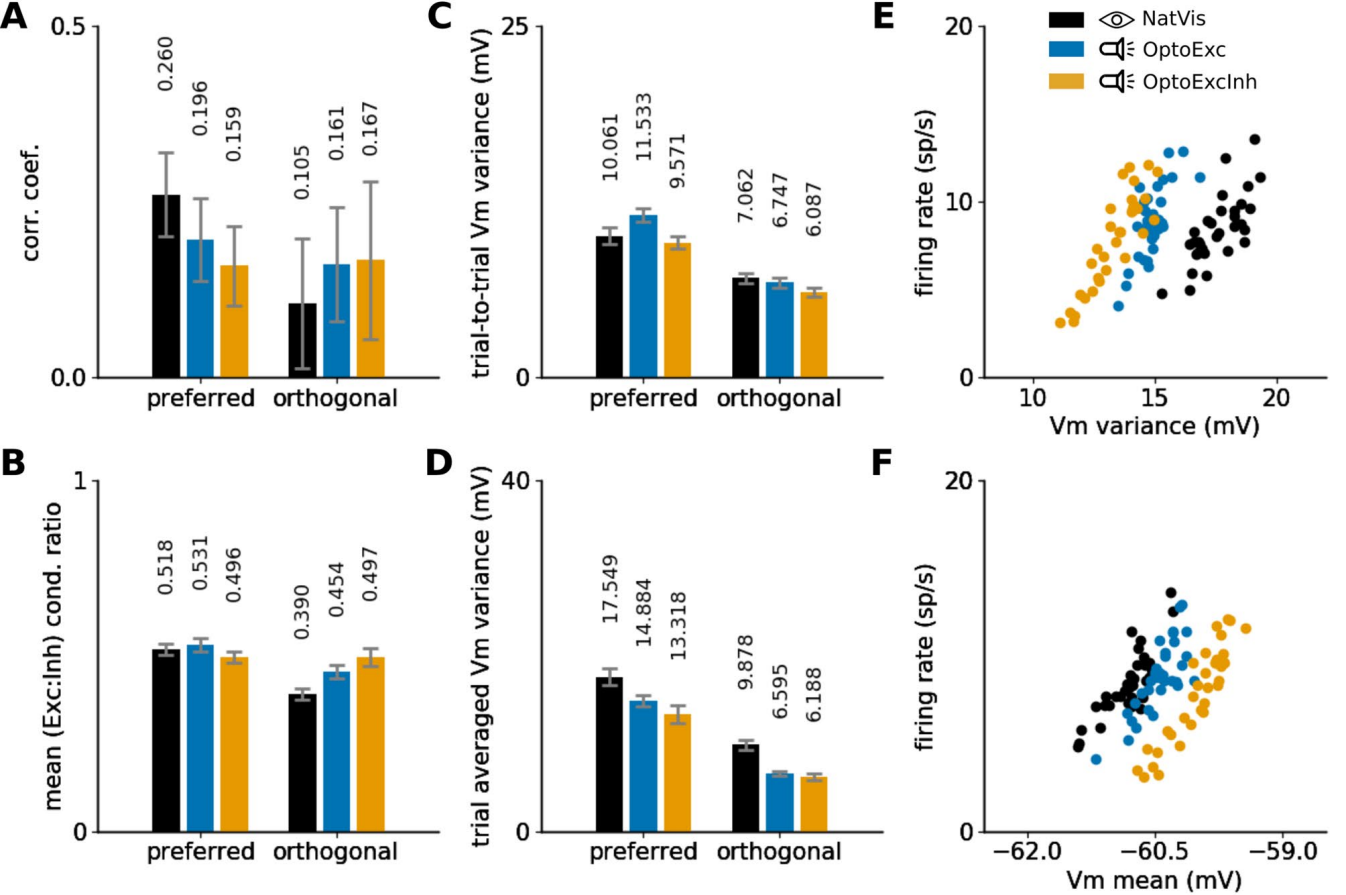
Naturally-driven versus optogenetically-driven response statistics. (**A**–**D**) Evoked statistics at the preferred (left bar group) and orthogonal (right bar group) orientations of the test grating in the three examined conditions: normal vision (black), light stimulation of excitatory cells only (blue) or both excitatory and inhibitory cells (orange). (**A**) Mean Pearson correlation between the PSTH (binned at 10 ms) of all pairs of recorded excitatory cells. (**B**) Ratio of mean excitatory and inhibitory conductance during grating presentation averaged across all recorded cells. (**C**) Mean trial-to-trial variability of the membrane potential during stimulation across the recorded cells. (**D**) Variability of $V_m$ averaged over the duration of the stimulus presentation and across all recorded cells. (**E** and **F**) Relationship between variance (**E**) and mean (**F**) of the membrane potential and the response rate of recorded excitatory cells at the preferred orientation.

Synchronization among neurons driven by the recurrent neural circuitry is an important driver of visual cortical response statistics^45^. We observe—that compared to the NatVis condition—synchronization is reduced in both OptoExc and OptoExcInh conditions (see Fig. 7A). This is expected, since intra-cortical processing up-stream of layer 2/3 is known to contribute to intracolumnar cortical synchronization.

#### E/I balance preserved across all conditions

We find that at the preferred orientation neurons across all three examined conditions show very similar levels in the excitatory to inhibitory (E/I) balance. This is surprising given the differences in the proportion of excitatory and inhibitory input to the layer 2/3 in the three examined conditions, further emphasizing that the recurrent cortical circuitry converges to a similar operating regime for a broad range of input drive scenarios. Furthermore, the NatVis condition exhibits greater drop (25%) of E/I balance when neurons are stimulated with preferred versus orthogonally oriented (non-preferred) grating compared to the OptoExc (16%) and OptoExcInh conditions (0%). This shows that the natural visual stimulation mediated by the feed-forward connections from layer 4 is more effective at recruiting lateral inhibition than direct external stimulation of layer 2/3.

#### Variance of the membrane potential

Both the trial-to-trial (Fig. 7C) and trial averaged (Fig. 7D) variance of the $V_{m}$ consistently decrease in neurons when they are stimulated by the orthogonal (non-preferred) stimulus in comparison to stimulation with preferred stimulus across all three examined conditions. For the trial-to-trial variance of the $V_{m}$, no clear differences across the examined conditions were observed. However, the trial averaged variance of $V_{m}$ is significantly higher for the NatVis condition than for the OptoExc and OptoExcInh conditions. This, however, raises a question. The variability of the membrane potential influences the mean firing rate of the neuron by influencing the likelihood of crossing the spike generation threshold^46,47^. Given that we ensure that the firing rates across the different conditions are matched to those of the NatVis condition (see Methods section "The stimulation strategy"), why is there a difference in variability of the $V_{m}$ between the NatVis and the two optogenetic (OptoExc, OptoExcInh) conditions? To elucidate this seeming discrepancy, we have further examined the relationship between the mean and variance of the $V_{m}$ and the firing rate of the response in all three examined conditions in the next section.

#### The dynamics regime of the OptoExc condition is closer to that of natural vision

As expected, the mean and variance of $V_{m}$ are positively correlated with the spike rate across all conditions (Fig. 7E,F). However, the underlying $V_{m}$ dynamics differ: the NatVis condition exhibits less tonic depolarization than the OptoExc and OptoExcInh conditions (Fig. 7F), but the NatVis condition compensates for the lower depolarization by higher variability of the $V_m$ to reach the same levels of response rates (Fig. 7E). Interestingly, the OptoExc condition exhibits dynamics closer to that evoked in the visually-driven condition than the OptoExcInh condition. Overall, these findings suggest that restricting the optogenetical transfection to excitatory cells is the preferred strategy for eliciting a naturalistic dynamical state in the stimulated cortex.

### Impact of the density of the MLEE on the induction of orientation tuning

In previous simulations we have assumed a MLEE (matrix of light-emitting elements) with a pitch and diameter of individual elements of 10 $\upmu$m, which is at the extreme of current technology. However, the smooth representation of visual features along cortical surface implies that a MLEE with coarser pitch could still be effective at functionally specific activation of V1.

**Figure 8:**
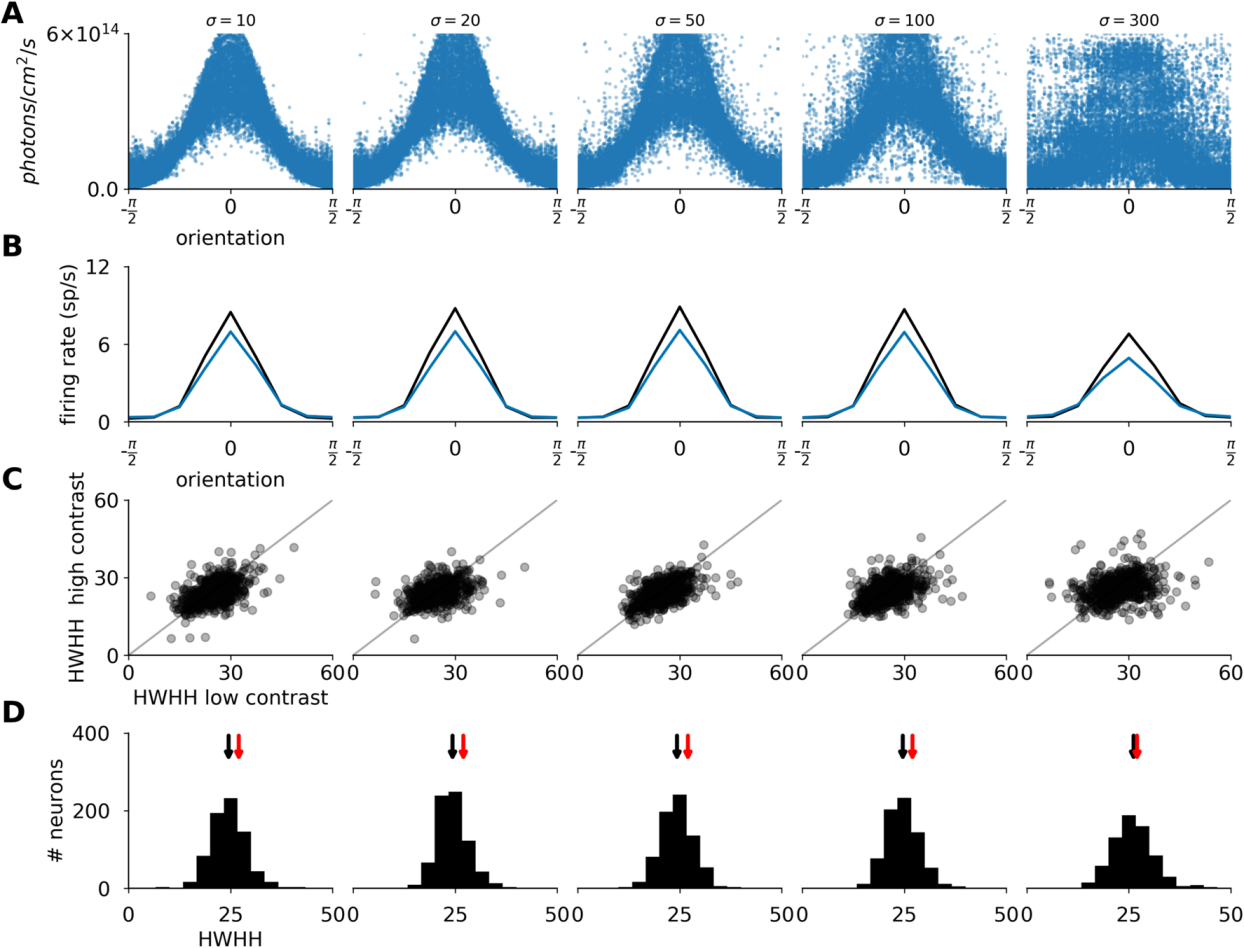
The impact of the diameter of individual light emitting elements (σ) on the induction of orientation tuning in V1. Each column corresponds to a cortical optogenetic stimulation with different size and pitch of light emitting elements in the OptoExc condition. (**A**) The illumination intensity at the cell body as a function of the orientation preference of the given neuron. (**B**) The orientation tuning curves centered and averaged across all recorded neurons. (**C**) The scatter plot showing the orientation tuning width measured as HWHH at low (abscissa) versus high (ordinate) contrast. (**D**) The histogram of tuning width measured as HWHH at maximum contrast. The black arrows on top mark the mean of the distribution. The red arrows mark the mean HWHH of the NatVis condition.

We have examined this issue in the OptoExc condition and find that optogenetic stimulation can accurately match the NatVis condition with a MLEE pitch of up to 100 $\upmu$m (Fig. 8B). For all tested conditions the proportion of neurons where the orientation tuning curve can not be well fitted by Gaussian remains below 4%, except for the 300 $\upmu$m size, where the ratio of neurons that are not well tuned increases to 11% (see section "The in-silico experimental protocols").

The contrast-invariance of the tuning is also largely maintained (Fig. 8C). Again, despite the fact that the orientation specificity of the illumination is degraded when increasing the individual light emitting element diameter (Fig. 8A), the intra-cortical connectivity is able to sharpen the illumination pattern greatly to produce orientation tunings matching those found during normal vision over a broad range of light stimulation element sizes (Fig. 8B). We find essentially identical results for the OptoExcInh condition (figure S7).

## Discussion

Surprisingly few computational studies have so far attempted to understand the impact of optogenetic stimulation on cortical dynamics^48,49^, and its implications for optogenetic encoding of visual stimuli in the cortex. To provide a further understanding of how functional neural networks interact with externally imposed optogenetic stimulation, we have simulated external optogenetic stimulation of a comprehensive spiking neural-network model of cat V1 using a matrix of light emitting elements. Our simulations show (1) that effective illumination intensity will depend on the cell-type specificity of ChR transfection (Fig. 4D), and (2) that extrapolating single cell results from in-vitro experiments that lack ongoing activity or network reverberations can lead to an over-estimation of the minimum amount of stimulation (threshold) required to evoke responses similar to those observed in-vivo, in line with recent modelling results^50^. We find a roughly 6-fold difference between the OptoDis condition (eT=3.0 $\times 10^{15}$ photons/s/cm$^2$) and OptoExc condition (eT=0.51 $\times 10^{15}$ photons/s/cm$^2$; Fig. 4D).

Next, our modelling shows that MLEEs with inter-element pitch as high as 100 $\upmu$m offer sufficient resolution for encoding elementary visual stimuli in cat cortex (Fig. 8). We also offer insights into how the visually driven and optogenetic evoked responses differ in terms of conductance activation patterns and membrane potential dynamics (Fig. 7). Finally, we have proposed and evaluated a stimulation protocol for induction of encoding of sinusoidal grating stimuli, forming a broad set of predictions that can help to interpret future experimental data. Gratings are canonical stimuli extensively used in vision science, and are thus an ideal target for initial prosthetic experiments in animal models.

We also address the question how the recurrent cortical interactions reshape the external stimulation pattern injected by the implant^44,49,51,52^. We find that intra-cortical interactions sharpen the resulting patterns of activity in comparison to the direct depolarizing drive imposed by the MLEE implant^44^. This is consistent with analogous experimental^53–55^ and theoretical^56,57^ findings for sensory-evoked depolarization. We suggest that this sharpening is the consequence of the broader mechanism of cortical attractor dynamics, which have been previously hypothesized to operate in V1^58,59^. The consequence of the intra-cortical recurrent circuitry implementing such attractor dynamics would be that the externally induced cortical activation patterns would be pushed towards cortical activation patterns that arise in response to natural visual stimuli.

These findings suggest that the intra-cortical interactions could mitigate some of the physical constraints of optogenetic light stimulation. We therefore propose that it might be more appropriate to design prosthetic stimulation protocols that mimic the biologically realistic distributed input pattern afferent to the targeted cortical volume, and let the cortical network self-organize to the drive, rather than individually clamping neurons at their desired output. This view is strengthened by a theoretical study of cortical dynamic attractors in asynchronous irregular networks by Frégnac and colleagues, which showed that reproducible spiking and subthreshold dynamics of the full network can be recovered if the statistics of the imposed external drive are consistent with the internal memories stored in the ongoing activity^60^. In this Gedanken experiment, a subset of neurons in the network, constrained to replay temporal pattern segments extracted from the recorded ongoing activity of the same network, was shown to reliably drive the remaining free-running neurons to recall the rest of the pattern. Accordingly, we propose here that the similarity between optogenetically-driven and visually-driven evoked activity in V1 could be improved, commensurate with the efficiency with which recurrency of the intracortical network can be recruited.

Our computational framework can contribute to screening of numerous aspects of potential stimulation strategies^50,61–63^, selecting only the most promising ones for future testing in animal models (once the prosthetic system is fully developed) and thus accelerating development and reducing animal experimentation. Several conclusions made in the present study, such as the predictions on the required spatial specificity for engaging stimulus orientation encoding in V1, can be extrapolated to neuro-prosthetic systems using other means of cortical stimulation.

Furthermore, understanding how neural encoding in V1 leads to the emergence of low-level (non-attentive) perception remains a hot topic in neuroscience (review in^64^). The combination of the high-fidelity modelling approach presented here with an optogenetic prosthesis could lead to a paradigm shift in how the neural basis of perception can be studied in animal models. The presented computational framework can help inspire innovative stimulation protocols that manipulate specific properties of cortical sensory coding, that are then tested in animal model using the visual prosthetic system, with the subsequent experimental measurements interpreted again within our modelling framework.

While the present paper focuses exclusively on how to utilize an optogenetic-based implant to encode visual information in V1, it is important to acknowledge the various unresolved challenges of such hypothetical prosthetic system. A major issue is the ability to engage targeted neurons with sufficiently strong and precise light stimulation with a small, wireless and implantable device. To that end, advances are being made at increasing the sensitivity of opsins^65^, increasing the power deliverable from MLEEs^66^ or alternatively miniaturization of two-photon microscopy^67^. Another issue, specific to primate cortex, is that only the cortical representation of the peripheral part of the visual field is accessible superficially under the skull, with the foveal representation largely hidden within the calcarine sulcus^32^. Developments of flexible bio-compatible material based implant technologies could in future allow for stimulation elements to be positioned inside the sulcus^68–71^, as well as lessen issues with mechanical movement of the implant^72^. Finally, while optogenetics has been developed in mice^21^, it has now been successfully validated in cat^73^, ferret^74^ and macaque^75^ V1, and there are ongoing clinical trials on application of optogenetics in human retina (ClinicalTrials.gov NCT02556736, NCT03293524, and NCT03326336).

Another major challenge is the need to identify the stimulus preference of neurons along the cortical surface under the implant, which in blind patients requires a protocol based on reported perceptual outcomes in response to activation of individual stimulation elements^2,76^. However, while in previous clinical trials only retinotopy had to be determined, our protocol requires the knowledge of the orientation preference representation as well. This could be potentially very time consuming, and further work will be necessary to make such a calibration process practical.

The presented V1 model, while parametrically constrained by the mammalian experimental literature, provides only a first-order emulation for simulating cortical blindness in humans. In particular, the planar approximation of the cortical sheet does not account for the natural folding of human cortex and the nonlinearity of the retino-cortical magnification factor. However, the choice of a non-primate model with similar functional organization to humans, such as cat, opens the possibility of implementing validation experiments for cortical visual prosthetics, before being applicable to human cortex where invasive multi-scale physiology is out-of-reach.

Finally, a particular concern is the impact of the transfected dendritic arbors on the spatial integration of light, which can effectively further reduce the spatial specificity of the light stimulation. Thus, simulations with morphologically detailed neurons should be performed in the future. Another limitation is that our proposed stimulation protocol does not accurately reproduce the transient dynamics at the onset and offset of the stimulus (see Fig. 4).

## Materials and methods

This manuscript relies on five key simulation components: (1) a stimulation strategy that translates a class of visual stimuli into driving signals for an MLEE, (2) a model of the matrix of light-emitting elements (MLEE), (3) a model of light propagation through cortical tissue, (4) a model of light illumination dependent ChR dynamics in transfected cells, and (5) a detailed large-scale model of primary visual cortex. As illustrated in Fig. 1, the combination of these components allows us to simulate the effects of light stimulation in a ChR transfected region of V1 given a specific stimulation strategy: the visual stimulus is translated by the stimulation strategy into a set of signals determining the level of activation of the individual light emitting elements. The light propagation model then determines the exact amount of light impinging onto individual neurons located in the simulated cortical volume. Next, simulating the ChR dynamics for each cortical neuron (given the amount of light it receives) determines the amount of current that is injected into the neuron. This finally allows us to simulate the dynamics of the simulated population of V1 neurons when embedded in the detailed, functionally specific V1 circuitry.

This simulation work has been based on our recent detailed large-scale model of primary visual cortex^33^, reproducing multi-scale behaviour of the V1 circuit, from conductance to spiking dynamics, for a wide spectrum of visual stimuli (ranging from gratings to animated natural scenes). The model has been implemented using the Mozaik neural simulation workflow framework^77^ and the Arkheia tool^78^. Here, we have extended the Mozaik framework with three additional components: the model of a MLEE, the model of light propagation in cortical tissue, and a model of ChR dynamics^79^. The NEST simulator^80^ was used as the back-end for all simulations described in this paper. In the remainder of this section we provide a detailed description of the new components, and a succinct description of the cortical model previously described elsewhere^33^.

### The model of light emitting elements and light propagation in cortical tissue

We assume a regular $5\times 5$ mm lattice (pitch 10 $\upmu$m) of circularly shaped light emitting elements of identical radius that is placed along the cortical surface and matches the laminar plane of supra-granular layers, approximating an LED or DMD matrix commonly used in optogenetic stimulation. As a first step, we determine the propagation of light through cortical tissue from a single light emitting element. We have performed the simulations using the Human Brain Grey Matter model implemented in the LightTools software, assuming 590 nm wavelength of the emitted light. The scattering and absorption properties of the human brain tissue are modelled using the Henyey-Greenstein model^81^. The two key parameters of this model are the anisotropy factor *g* and mean free path (MFP) which are both dependent on wavelength. Considering the 590 nm wavelength we have set the two parameters to 0.87 and 0.07 mm based on Jacques et al.^81^. It should be noted that the accuracy and current knowledge of biological optical properties of cortical matter is limited and both the inter and intra sample variability has been reported to be as much as 30%.

This way we obtain a 2D table *T*(*d*, *l*) capturing the light flux (photons/s/cm$^2$) in cortical tissue relative to the value at the surface of the light emitting element as a function of depth *d* and the lateral distance (along the cortical surface) from the light source *l*. The light flux at the location of the given neuron *n* in the cortical volume is then calculated as a linear sum of the contributions from the individual elements in the matrix:

1 $$\begin{aligned} \gamma _{n} = \sum _{e} \beta _e T(d_n, \Vert c_e - c_n \Vert ) \end{aligned}$$

where $\gamma _{n}$ is the resulting light flux at neuron *n*, $\beta _e$ is the light flux at the surface of the light element *e*, and $c_e$ and $c_n$ are the lateral coordinates along the cortical surface of element *e* and neuron *n* respectively.

### The channelrhodopsin model

We have used the model of ChrimsonR channel dynamics recently implemented by Sabatier et al.^79^. The electro-chemical behaviour of the ChrimsonR protein is modelled using a Markov kinetic model^82^. In this model, five states represent the different conformations that the protein can take. For each pair of states, there can be a directed transition from one state to the other if there exists a chemical switch from the first state to the second. A time constant is associated with each transition. A transition can either be thermal or photo-induced. Thermal transitions have fixed time constants, while photo-induced transition’s time constants vary with the current intensity of the light stimulus. A photo-induced reaction cannot occur in the absence of light.

Mathematically, the values of the transition time constants along with the light stimulus describe the linear differential system governing the evolution of the proportion of channels (or equivalently the probability for a single channel) in each state. The relevant figure, the conductance of the population of channels in a single neuron, is then derived from the number of channels in the open states and the conductances of these states.

The parameters of this model have been fitted by Sabatier et al.^79^ to light (590nm wavelength) stimulation experiments in ChrimsonR-expressing HEK293 cells, and here we use the parameter values reported in this study.

### The stimulation strategy

Sinusoidal grating stimuli are classical Fourier inputs used to characterize the global transfer function of the visual system, both electrophysiologically and behaviourally^83^. Below we present a optogenetic stimulation protocol that can impose cortical responses similar to those evoked by grating stimuli during normal vision. To do so quantitatively, we need to take into consideration several physical and biological constraints.

Due to the absorption and dispersion of the light in cortical tissue, the intensity and resolution (contrast) of the pattern of light induced by the MLEE degrade with increasing cortical depth (see section "The model of light emitting elements and light propagation in cortical tissue"). Because cortical layer 2/3 is closer to the surface and at the same time sends its output towards higher cortical areas^84^, it is a suitable target for optogenetic based intervention for vision restoration.

The majority of neurons in V1 are selective to the orientation of the stimulus^43^. Across the cortical surface, the functional orientation preference assembly of V1 neurons is topologically organized into smooth iso-preference orientation domains (Fig. 3). Layer 2/3 is primarily populated with the non-linear complex cell type^34,35^, which is invariant to the precise position of the stimulus in its receptive field (RF), thus responding by a tonic elevation of their membrane potential and spike response to a grating drifting across its RF. Taking into consideration all the above constraints, we propose the following stimulation strategy:

1. Assuming the prior knowledge of the local orientation preference map in the targeted cortical volume, assign the orientation preference $OR_M$ to each light emitting element *M* located at the cortical surface coordinates $C_M$ as the weighted average of orientation preferences of individual neurons sampled in the neighbourhood centered at $C_M$ (Fig. 3A).
2. For a full-field sinusoidal grating of orientation $\rho$ and each light emitting element *M*, calculate the orientation dependent activation index $\psi _{\rho ,M}$ as $\psi _{\rho ,M} = f(\delta (\rho ,C_M))$, where $\delta$ is the circular distance and *f* is a function of distance. In this study we set *f* to a Gaussian function with zero mean and $\sigma =0.5$ variance (except in section "Optogenetically induced orientation tuning of cortical responses" where $\sigma$ is varied; Fig. 6A).
3. Set the signal driving light emitting element *M* as a step function such that the resulting light output at its surface (photon flux measured in photons/s/cm$^2$) is $\theta _M = S_{M}(\tau _s,\tau _e,\phi )$, where $\tau _s$ is the start of the step and the $\tau _e=\tau _s + d$ is the end of the step, where *d* is the duration of the grating stimulus (Fig. 3B). The $\phi = L_{max} \psi _{\rho ,M}$ is the magnitude of the step, where $L_{max}$ is an overall stimulation scaling factor setting the maximum light emission at the surface of the MLEE.

The $L_{max}$ is an arbitrary scaling factor that has to be determined experimentally, and encompasses scaling unknowns such as the rate of ChR transfection, or light absorption in the cortex. It also takes into account stimulus dependent scaling. In supplementary section 1.2, we present a method for determination of the $L_{max}$ parameter for grating stimuli. In principle, the $L_{max}$ also depends on other parameters of V1 selectivity, such as spatial or temporal frequency, but for the sake of simplicity, in this study, we assume that the other parameters are kept at the known preferred values at the given retinotopic eccentricity of the targeted V1 volume.

### The large-scale spiking model of primary visual cortex

This model is derived from the full model presented in Antolik et al.^33^. The cortical model corresponds to layers 4 and 2/3 of a 5$\times$5 mm patch of cat primary visual cortex. Given the magnification factor of 1 at 5 degrees of visual field eccentricity^85^, the visual span covers roughly 5$\times$5 degrees of visual field. To avoid edge effects, the centres of LGN model neurons span 7.5$\times$7.5 degrees of visual field, and the total visual field in which the stimulus was presented to the model spans 10$\times$10 degrees. The model contains 30625 neurons and $\sim 30$ million synapses. This represents a significant down-sampling ($\sim$10%) of the actual density of neurons present in the corresponding portion of cat cortex^86^ and has been chosen to make the simulations computationally feasible. Each simulated cortical layer contains one population of excitatory neurons (corresponding to spiny stellate neurons in Layer 4 and pyramidal neurons in Layer 2/3) and one population of inhibitory neurons (representing all subtypes of inhibitory interneurons) in the ratio 4:1^87,88^.

We restrict the model to monocular input and do not model any topological organization of binocularity in V1—all neurons respond to the single modelled retina. The thalamic input reaches both excitatory and inhibitory neurons in Layer 4 (see Fig. 2E,F). In both cortical layers, we implement short-range lateral connectivity between both excitatory and inhibitory neurons. Additionally, in Layer 2/3, we also model long-range excitatory connections onto other excitatory and inhibitory neurons^89–91^ (see Fig. 2A,B). Layer 4 excitatory neurons send narrow projections to Layer 2/3 neurons (see Fig. 2E). The model omits the infra-granular layer 5 and 6 as well as the cortical feedback to perigeniculate nucleus (PGN) and LGN.

We have validated the functional features of the model that are critical for the present study against the experimental literature in supplementary section 1.1. Particularly, we verified the asynchronous irregular spontaneous dynamical regime, the emergence of contrast-invariant orientation tuning across the model cortical layers, and the presence of simple/complex cell types across the two modelled cortical layers. A detailed description of the model construction follows.

#### Neuron model

All neurons were modeled as the exponential integrate-and-fire units (Eq. 2), whereby the time course of the membrane potential $V_m$ is governed by:

2 $$\begin{aligned} \tau _{\mathrm{m}} \frac{\mathrm {d}V_{m}}{\mathrm {d}t} = - \left( V_{m}- V_{\mathrm{rest}}\right) + \frac{\varDelta T}{R_{\mathrm{m}}}\mathrm {exp}\left( \frac{V_{m}-V_{T}}{\varDelta T}\right) + R_{\mathrm{m}}g_{\mathrm{exc}}(E_{\mathrm{exc}} - V_{m}) + R_{\mathrm{m}}g_{\mathrm{inh}}(E_{\mathrm{inh}} - V_{m}) \end{aligned}$$

where $g_{\mathrm{exc}}$ and $g_{\mathrm{inh}}$ are the incoming excitatory and inhibitory synaptic conductances. The emission time of spikes is registered when the membrane potential crosses the 0 mV threshold, at which time the membrane potential is set to the reset value $V_{\mathrm{r}}$.

#### Connectivity

All neurons in the model Layer 4 receive connections from the model LGN (see section "Input model"). For each neuron, the spatial pattern of thalamo-cortical connectivity was determined by a Gabor distribution, inducing the elementary RF properties in Layer 4 neurons^92,93^ (see Fig. 2E,F). A pre-computed orientation map was overlaid onto the modelled cortical surface, thereby assigning each neuron an orientation preference. The remaining parameters of the Gabor were set to constant values, matching the average of measurements in cat V1 RFs located in the para-foveal area^94,95^.

Each excitatory neuron received 1480 synaptic inputs (for detailed justification please refer to Antolik et al.^33^). Inhibitory neurons received 30% fewer synapses than excitatory neurons to account for their smaller size. 35% of synapses from Layer 4 cells were formed on the Layer 2/3 neurons. In addition, layer 4 cells received on average 10 additional thalamo-cortical synapses^96^. The synapses were drawn probabilistically with replacement (with functional and geometrical biases described below).

The geometry of the cortico-cortical connectivity was determined based on two principles: the connection probability falls off with increasing cortical distance between neurons^90,97,98^ (see Fig. 2A,B), and connections have a functionally specific bias^90,99^. The two principles were each expressed as a connection-probability density function, then multiplied and renormalized to obtain the final connection probability profiles, from which the actual cortico-cortical synapses were drawn.

The spatial extent of the model local connectivity, with the exception of excitatory lateral connections in Layer 2/3, were established based on a re-analysis of data from cat published in Stepanyants et al.^98^. For details of this analysis and resulting parameter values please refer to Antolik et al.^33^.

With respect to functional bias, within Layer 4 we assumed push-pull connectivity^92^ (see Fig. 2E). For each pair of Layer 4 neurons the correlation *c* between their afferent RFs was calculated. The connectivity likelihood for a given pair of neurons is given by $\frac{1}{{\sigma \sqrt{2\pi } }}e^{{{ - \left( {c-\mu } \right) ^2 } \big / {2\sigma ^2 }}}$ where $\sigma =1.4$ and $\mu$ is 1 if the pre-synaptic neuron is excitatory and $\sigma =3.0$ and $\mu$ is -1 if the pre-synaptic neuron is inhibitory.

To reflect the long-range orientation biased connectivity in layer 2/3 that is absent in layer 4, we have defined the connectivity likelihood between pairs of neurons in Layer 2/3 as $\frac{1}{{\sigma \sqrt{2\pi } }}e^{{{ - \left( {\Delta o} \right) ^2 } \big / {2\sigma ^2 }}}$ where the $\Delta o$ is the difference between the orientation preference of the two neurons, and $\sigma$ was set to 1.4 for excitatory neurons and to 3.0 for inhibitory neurons.

Finally, apart from the connectivity directly derived from experimental data, we have also considered a direct feedback pathway from layer 2/3 to layer 4. Such direct connections from layer 4 to layer 2/3 are rare^100^, however a strong feedback from layer 2/3 reaching layer 4 via layers 5 and 6 exists^100^. Please refer to Antolik et al.^33^ for detailed justification of the proposed connectivity scheme.

#### Synapses and delays

Synaptic inputs were modeled as transient conductance changes with exponential decay with time-constant $\tau _e=1.1$ ms for excitatory synapses and $\tau _i=1.9$ ms for inhibitory synapses. We have set the unitary synaptic weight of all cortico-cortical excitatory to inhibitory synapses to 1.3 nS. All other cortico-cortical synapses have been set to 0.8 nS. Futhermore, thalamo-cortical synapses were set to be slightly stronger, reflecting their higher reliability and larger size: 1.2 nS onto excitatory neurons, 1.44 nS onto inhibitory neurons. We have also modeled synaptic depression for thalamo-cortical, and excitatory cortico-cortical synapses^101^ using the model of Markram et al.^102^.

We model two types of delays in the model circuitry. First, the delays due to the distance dependent propagation (horizontal propagation constant of $0.3\,\hbox {ms}^{-1}$^103–105^) were considered. The delays in the feed-forward thalamo-cortical pathway are drawn from a uniform distribution within the [1.4, 2.4] ms range. Second, Ohana et al.^106^ have shown that delays of synaptic transmission in cat visual cortex are dependent on both pre- and post-synaptic neural type, with the notable feature of slow excitatory to excitatory, and fast excitatory to inhibitory transmission. Thus we included a constant additive factor in all synaptic delays, specifically 1.4 ms for excitatory to excitatory synapses, 0.5 ms for excitatory to inhibitory synapses, 1.0 ms for inhibitory to excitatory synapses, and 1.4 ms for inhibitory to inhibitory synapses^106^. These delay factors stabilized the simulation by reducing synchronous events during spontaneous activity.

#### Input model

The input model treats the retina and thalamus as a single layer integration stage. We use the widely-accepted center-surround model of receptive fields (RFs) to simulate the responses of the LGN neurons (Fig. 2C). The centers of both ON- and OFF-center LGN neurons RFs are uniformly randomly distributed in the visual space, with a density of 100 neurons per square degree. Each LGN neuron has a spatiotemporal RF, with a difference-of-Gaussians spatial profile, and a bi-phasic temporal profile defined by a difference-of-Gamma-functions. Due to the relatively small region of visual space our model covers, we do not model the systematic changes in RF parameters with foveal eccentricity. The exact spatial and temporal parameters were taken from Allen and Freeman^38^.

To obtain the spiking output of a given LGN neuron, the visual stimulus was sampled into 7 ms frames, and convolved with its spatiotemporal RF. In addition, we modelled saturation of the LGN responses with respect to local contrast and luminance^107,108^ (refer to Antolik et al. for details^33^). The resulting temporal traces are then summed and injected into integrate-and-fire neurons as a current, inducing stimulus dependent spiking responses. Additionally, neurons are injected with white noise current. The magnitude and variance of this noise is such that neurons fire spontaneously at the rate of $\sim 10$ spikes/s^92,109^.

### The in-silico experimental protocols

Monocular full-field sinusoidal grating stimuli of varying orientation drifting at 2 Hz were used to probe the canonical functional property of V1 neurons: orientation preference and tuning selectivity. We used two variants of this protocol to simulate natural sensory dynamics evoked through intact retina and cortical dynamics imposed through prosthetic vision by optogenetic light stimulation (see section "The stimulation strategy"). The sinusoidal gratings were presented at 8 equally spaced orientations around the circle. Each grating was shown 10 times for 600 ms. The spatial and temporal frequency of the RFs of the modeled LGN neurons and of the Gabor distribution template from which thalamo-cortical synapses were sampled were identical. Therefore, by employing a full-field stimulus with spatial and temporal frequency matching the selectivity of afferent cortical RFs, we were able to co-stimulate optimally all cortical neurons selective to the given orientation. The sanity of the model of the prosthetic device, light propagation through cortex, and ChR dynamics have been validated using the decoupled model (see supplementary section 1.1).

The section "The stimulation strategy" describes light stimulation protocol for evocation of cortical activity corresponding to visual stimulation by specific full-field sinusoidal grating stimulus. The prosthetic vision variant of the orientation tuning protocol followed the same series of corresponding stimuli as described in the previous paragraph, rendering the evoked responses directly comparable between the two orientation tuning protocol variants.

In order to assess orientation tuning of the observed responses, we calculated the HWHH measured by fitting the orientation tuning curves with a Gaussian function^42,110^:

3 $$\begin{aligned} R(\phi ) = \beta + \alpha \exp \left( \frac{\phi - \phi _{\mathrm{pref}}}{2\sigma ^2}\right) \end{aligned}$$

where *R* is the spiking response of the given neuron to a sinusoidal grating with orientation $\phi$, $\phi _{\mathrm{pref}}$ is the preferred orientation of the given neuron, $\sigma$ is the width of the tuning, $\beta$ is the baseline activity, and $\alpha$ a scale factor. Neurons for which a reliable fit of a Gaussian curve was not possible ($MSE > 30\%$ of the tuning curve variance) were also excluded from this analysis. HWHH was then calculated as .

### Data analysis

In Fig. 4D we fit the relationship between the photon flux at the cell body and the response of the cell with a sigmoid:

4 $$\begin{aligned} R(F) = S \frac{1}{1+e^{-G(F-T)}} \end{aligned}$$

*R*(*F*) is the response at photon flux *F*, *S* is the scaler factor, *G* is the gain, and *T* is the threshold parameter. The threshold parameter *T* of the sigmoid function does not reflect the threshold of the stimulus-response relationship well, which is typically defined in an experimental setting as the point where the response function departs significantly from the spontaneous rate. To obtain a comparable index we calculate what we will refer to as an effective threshold (eT), which is the photon-flux for which the fitted sigmoid reaches $5\%$ of its asymptotic maximum. It should be noted that the choice of $5\%$ here is arbitrary, and slightly different values would result in a systematic shift across all conditions of the eT estimate. However, in this study we will use the eT parameter only to make comparisons between conditions which remain valid.

### Choice of animal model

Neural prosthetic systems are first tested in animal models. We have chosen to base our model on the cat as the most extensive body of data on primary visual cortex has been collected in this species. The relevance to human physiology of the columnar cortical organization, best documented in cat and non-human primate, has been well confirmed using more mesoscopic structural measures or quantitative fMRI^111,112^. Several of the most important datasets for this study, such as extensive parametric studies of visual receptive fields, the extents of inter-areal connectivity in V1^98^, and the relative strength of the different intra-areal pathways^100^ has been collected in this species. Finally this animal model is the only species where neural data have been obtained from the microscopic range (using intracellular current or voltage clamp recordings) to the mesoscopic range (MUA, LFP, VSD), and for a wide range of visual input statistics ranging from sparse noise and gratings to animated natural scenes. These data have served to constrain a realistic multi-scale model of cat V1 which is the core of the simulations presented in this article.

## Supplementary information

Supplementary Information

## Data Availability

An exact, machine readable specification of the model and experimental protocols can be found at http://corticalprosthesismodel.arkheia.org/. The raw virtual recordings generated during the current study are available from the corresponding authors on reasonable request.
