# Supplementary material for: Assessment of optogenetically-driven strategies for prosthetic restoration of cortical vision in large-scale neural simulation of V1
Source: Sci Rep. 2021 May 24;11:10783. doi: 10.1038/s41598-021-88960-8 (PMC8144184; doi:10.1038/s41598-021-88960-8)
Supplement: Supplementary file 1 — Supplementary Information [file 41598_2021_88960_MOESM1_ESM.pdf]

Assessment of optogenetically-driven strategies for prosthetic restoration of cortical vision in large-scale neural simulation of V1

Jan Antolik[1,2,⋆], Quentin Sabatier[2], Charlie Galle[3], Yves Frégnac[4], and Ryad Benosman[2,5,⋆]

[1]Faculty of Mathematics and Physics, Charles University, Malostranské nám. 25, 118 00, Prague 1, Czechia
[2]INSERM UMRI S 968; Sorbonne Universits, UPMC Univ Paris 06, UMR S 968; CNRS, UMR 7210, Institut de la Vision, F-75012, Paris, France
[3]Gensight Biologics, 74 Rue Faubourg Saint-Antoine, Paris, France
[4]Unité de Neurosciences, Information et Complexité (UNIC), NeuroPSI, Gif-sur-Yvette, France
[5]University of Pittsburgh Medical Center, Biomedical Science Tower 3, Fifth Avenue, Pittsburgh, PA, USA
[⋆]corresponding author

November 23, 2020

1 Supporting Information

1.1 Validation of functional properties of the V1 model

The cortical V1 model used in the present study depends on the presence of following functional features in the large-scale model of the primary visual cortex: (i) a realistic on-going regime, (ii) a realistic orientation tuning of V1 neurons, and (iii) a realistic distribution of simple vs. complex neural types across layer 4 and 2/3. In this section we validate that all these three features are present in the model and quantitatively match experimental data.

1.1.1 Background state

The spontaneous activity of excitatory neurons in both cortical layers (4 and 2/3) can be characterized as asynchronous (figure S1D: the mean cross-correlation of the spike counts in excitatory populations is less then 0.02) and irregular (CV of ISI greater than 0.8 in both populations (see figure S1C)). This is consistent with experimentally identified ongoing regime in cortical slices [1, 2], awake animals [3, 4] and up-states in in-vivo anesthetized preparations [3, 5]. Mean spontaneous rates of inhibitory neurons is greater than in excitatory ones, in line with experimental evidence [6, 7], and the spontaneous rates of most excitatory neurons is low (<2 Hz), as observed in cat V1 [8, 9] (figure S1A). Next, we observe slightly more depolarized resting membrane potential across all four cortical populations (figure S1D) than the typical resting state of about -70 mV observed in in-vivo V1 recordings in anaesthetized cat [10]. Finally, the distribution of firing rates in the model follows log-normal distribution (Figure S1E-H), as previously shown in different cortical areas and species [11, 12].

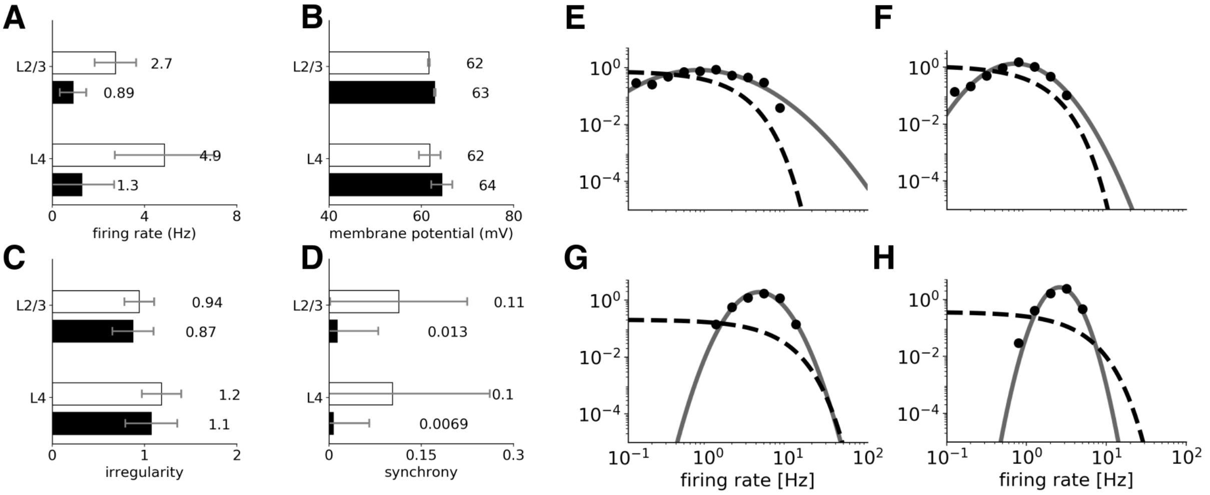

Figure S1: Statistics of spontaneous cortical regime. (A–D) Measures of spontaneous activity for excitatory (black bars) and inhibitory (white bars) neural populations in the two modelled cortical layers. Error bars report standard deviation. (A) Mean single-unit firing rates. (B) Mean membrane potential. (C) Irregularity of single-unit spike trains quantified by the coefficient of variation of the inter-spike intervals. (D) Synchrony of multi-unit spiking activity quantified as the mean correlation coefficient between the PSTH (bin size 10 ms) of all pairs of recorded neurons in a given population. (E–H) The distribution of firing rates (black points) in Layer 4 excitatory (E), Layer 2/3 excitatory (F), Layer 4 inhibitory (G), and Layer 2/3 inhibitory (H) neurons is well fitted by a log-normal distribution with matching mean and variance (gray line, log-log scale). The data is better fit by a log-normal (gray line) than by an exponential (dashed line) distribution.

1.1.2 Orientation tuning

We have measured orientation tuning of neurons in all four cortical populations (layer 4 excitatory: 24.22°, layer 4 inhibitory: 29.47°, layer 2/3 excitatory: 25.47°, and layer 2/3 inhibitory: 39.41°) using

sinusoidal drifting gratings evenly spanning the orientation circle at 8 intervals shown for 2 seconds and 10 trials each. Neurons in all four cortical populations are well orientation tuned. The mean orientation tuning measured as HWHH of all four populations (layer 4 excitatory: , layer 4 inhibitory: , layer 2/3 excitatory:, and layer 2/3 inhibitory:) is within the range outlined by previous experimental studies in cat [13, 14, 15]. Furthermore, in line with [13], when measured as HWHH, the orientation tuning of inhibitory populations is on average slightly broader than that of excitatory population in both layer 4 and 2/3, but crucially inhibitory neurons remain well tuned. Finally, in all cortical model populations the mean absolute difference between HWHH at low (5%) and high (100%) contrast was less then 10% (layer 4 excitatory: 2.28°, layer 4 inhibitory: 1.46°, layer 2/3 excitatory: 1.27°, and layer 2/3 inhibitory: 2.48°), indicating the emergence of contrast invariant orientation tuning in the model V1.

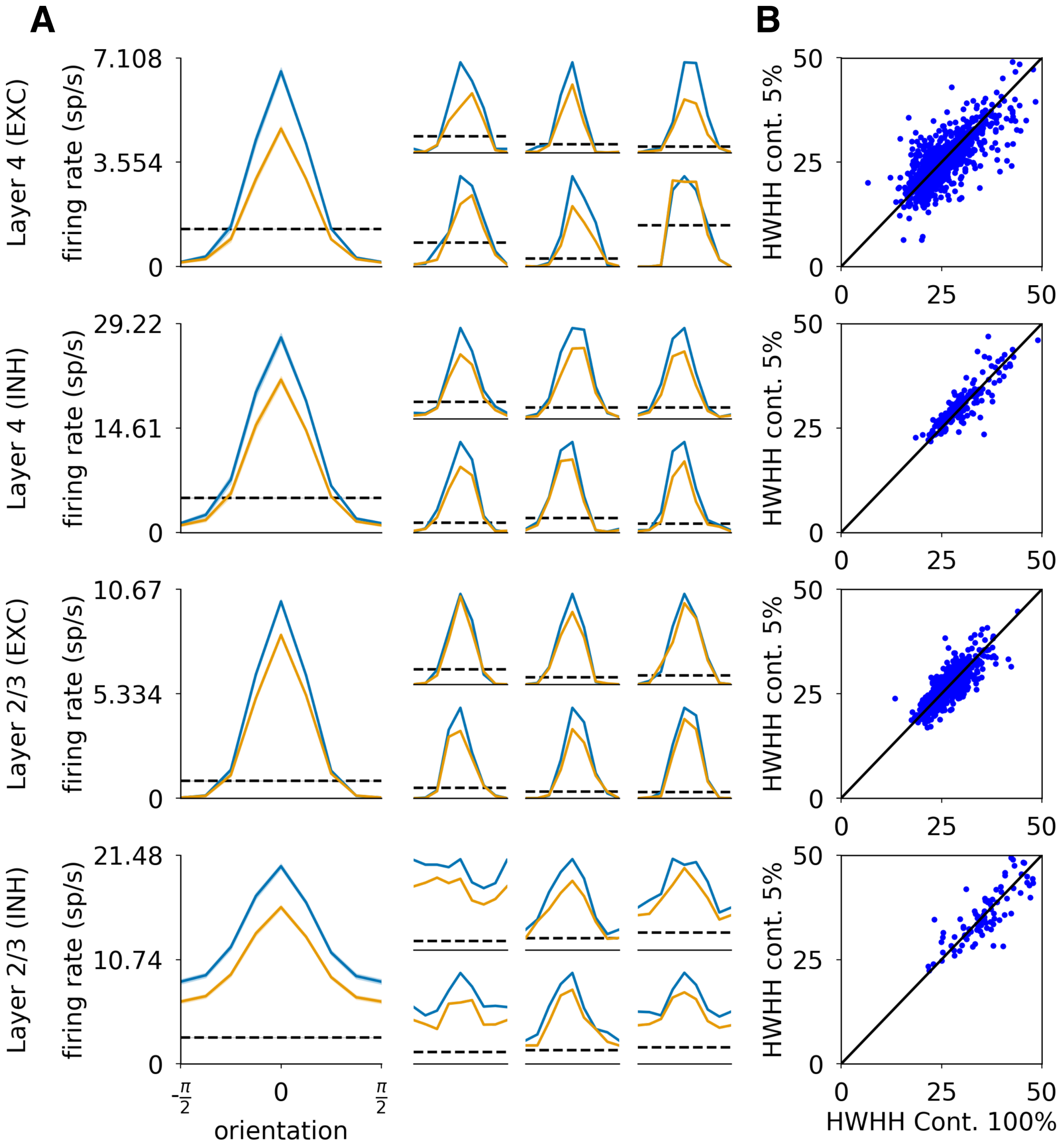

Figure S2: Orientation tuning in the four cortical model populations. (A) Mean orientation tuning across all cells in the given population (left larger panel), and six single cell examples (6 smaller right panels). (B) Scatter plot of the HWHH of orientation tuning at low vs. hight contrast.

1.1.3 Modulation Ratio

We have measured the modulation ratio (as the ratio of the first harmonic and mean of the firing rate) of spiking response of cortical excitatory cells. As shown in figure S3, majority of neurons in layer 4 have $MR > 1$ and thus belong to the simple cell category, while majority of neurons in layer 2/3 have $MR < 1$ and thus belong to the complex category.

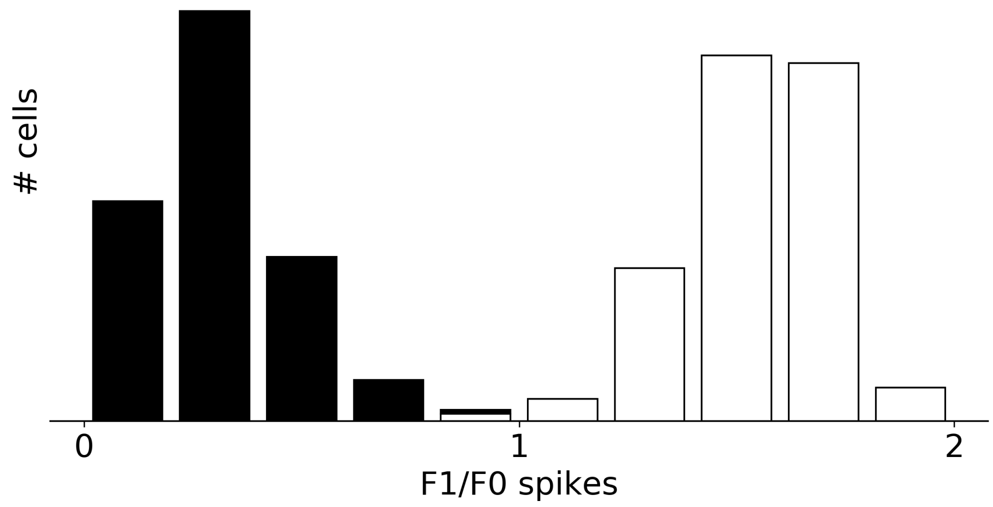

Figure S3: Modulation ratio of spiking response in model layer 4 and layer 2/3. Layer 4 cells are shown in white, and layer 2/3 cells in black.

1.2 Benchmarking the optogenetic-based cortical stimulation simulation

As a sanity of the optogenetic-based cortical prosthetic system simulation, we examined a simplified version of the model where all intra-cortical connections have been disabled. Thus the neurons membrane potential is fully determined by two factors: (i) position in the cortical volume and (ii) the light stimulation. As expected, the stimulation protocol (section 4.3) elicits step-like inward currents in layer 2/3 (figure S4B) neurons that bear the characteristics of the ChR dynamics (see Methods), and that are further transformed in the cell's membrane potential (figure S4A). Increased intensity of light (L_{max}) induces increased ChR mediated inward currents (figure S4E), hence more depolarized membrane-potential, and once the action potential threshold is crossed, firing rate becomes correlated with light intensity (figure S4CD). Furthermore, as the cortical depth increases, neural response decreases (figure S4CF) due to the attenuation of incoming light as a result of its absorption and dispersion in neural substrate. Increasing depth also delays the onset time of the response due to the reduced driving force causing slower increase from resting to threshold membrane potential (figure S4C). Finally, figure S4G shows the magnitude of photon flux as a function of the orientation of the neuron, demonstrating the expected orientation dependence of the stimulation protocol. Finally, we would like to note, that all the variations in figure S4F and G are due to the variations of neuron's depth or lateral displacement of neurons with respect to the orientation map features (see Methods).

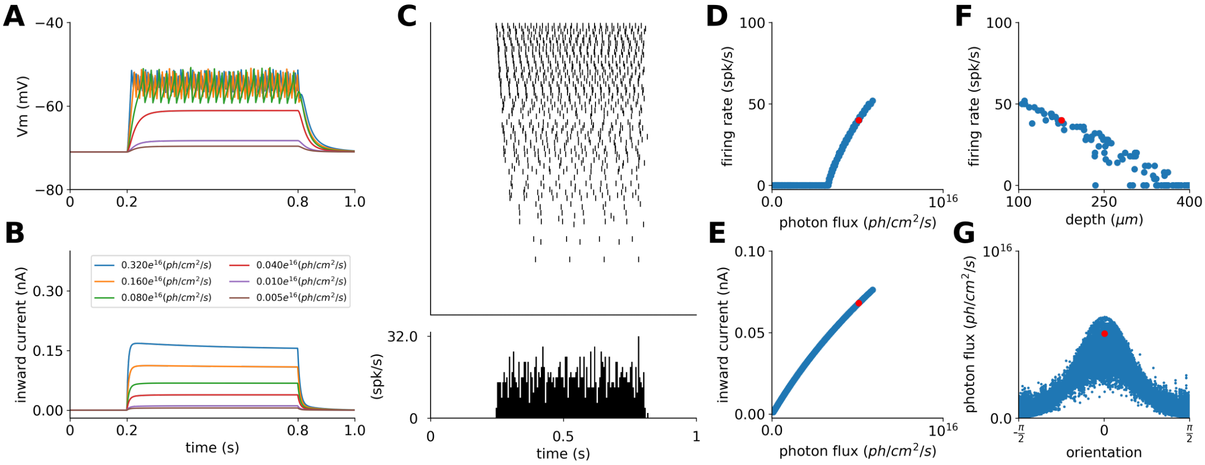

Figure S4: Light stimulation in cortical model with disabled connections. (A) The membrane potential of a representative cell at different levels of intensity of light impinging on the cell (the color coding corresponds to panel B). The light stimulation was a step function, starting at 200ms and ending at 800ms. (B) The inward current elicited by the light stimulation of different intensities (the legend shows the maximum photon-flux at the surface of the MLEE) in the same cell as in A. (C) The spiking response of population of cells recorded in central region of the model in response to light stimulation. The cells were ordered according to their cortical depth increasing from top to bottom. (D) The relationship between the photon flux at the position of neurons in the cortex and their firing rate. (E) The relationship between the photon flux at the position of neurons in the cortex and the resulting inward current. (F) The relationship between the depth of neurons in the model cortical substrate, and their response. (G) The relationship between orientation preference of neurons (abscissa) and their firing-rate response (ordinate) to light stimulation emulating sinusoidal grating of $\frac{\pi}{2}$ orientation. The stimulation followed the strategy presented in section 4.3. Neuron depicted in A and B was marked in red in D,E,F,G. With the exception of (G) only neurons whose orientation preference matched with the orientation of the light-stimulation emulated grating stimulus were considered in this analysis.

1.3 Calibration of contrast response curves

For the purpose of effective prosthetic stimulation protocol, and to facilitate better comparison between optogenetically- and visually-driven conditions, we need to match the magnitude of response due to optogenetic stimulation to physiological levels expected for the given visual stimulus. During normal vision, the magnitude of the depolarization due to grating stimulus in complex cells grows with stimulus contrast. In the optogenetically-driven stimulation protocol the analogue of the contrast parameter is the light intensity scaling parameter L_{max} (Methods 4.2). In the following, we devise a scheme for matching the light intensity with the contrast of the visual stimulus, such as to obtain a similar magnitude of response.

The contrast-response curve of V1 neurons fits the Naka-Rushton function well [15].

$$R(x) = R_{max}\frac{x^n}{x^n + K^n} \tag{1}$$

where $R(x)$ is the response at either contrast level x or stimulation light intensity x, R_{max} is the asymptotic maximum response amplitude, K is the semi-saturation constant, and n controls the slope. Conveniently, the Naka-Rushton function also fits well the relationship between the light intensity and evoked firing rates in the 3 optogenetic stimulation conditions (figure S5A,B,C).

As seen with in-vivo neurons, we observe in our simulations considerable variability of the fitted parameters from cell to cell. Due to the physical constraints of current implant technology, it is impossible to control the activity of each individual neuron independently. Therefore, we aim to match the mean contrast-response curve across local cortical volume. Having the parametric fits of the light-to-response tuning curve F and contrast-to-response tuning curve G, this can be achieved by mapping the desired contrast onto rate using G and then mapping rate using inversion of F on the light intensity (figure S5D). Figure S5D shows that this method leads to a good match of the mean contrast response curves between the visually-driven and optogenetically-driven conditions. We will be using this method in the remainder of the study to perform contrast matched comparisons across conditions.

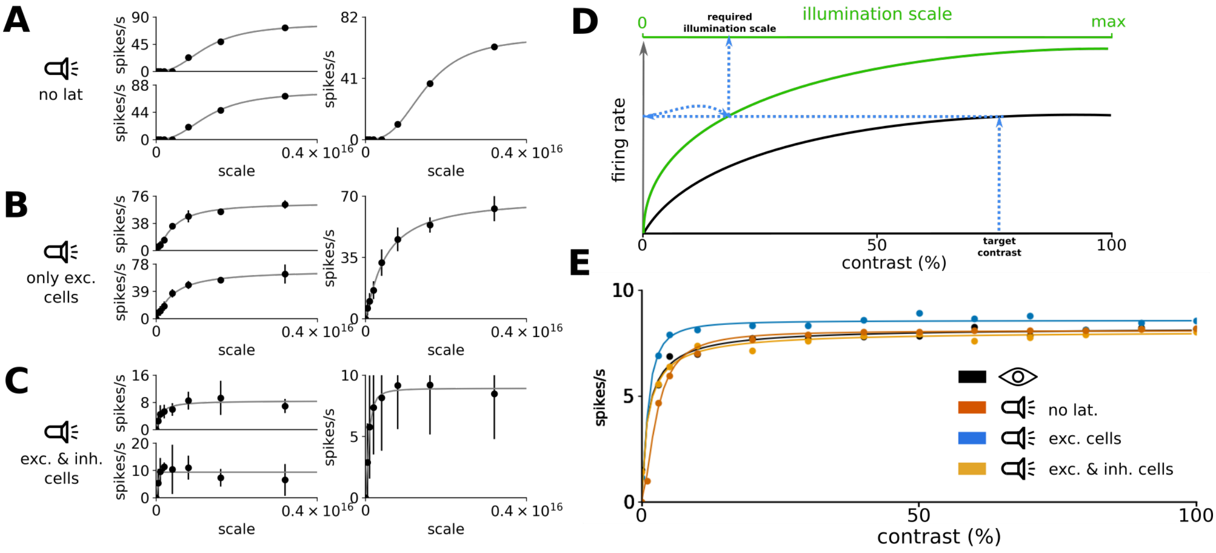

Figure S5: Contrast to stimulation light intensity mapping. (A-C) The stimulation light to response curve of two example individual neurons (left panel) and the mean curve over all recorded neurons (right panel). Three conditions are shown: no intra-cortical connectivity (A, uncoupled), only-excitatory neurons excitable by light (B) and both excitatory and inhibitory neurons excitable by light (C). (D) A scheme for matching visual stimulus contrast to the optogenetic stimulation intensity that will induce the same level of response as presentation of the stimulus under normal vision. (E) The resulting contrast-response curves of the three optogenetic conditions superimposed over the normal vision data. In all panels, the dots are data points from simulations, line is the Naka-Rushton curve fit.

1.4 Orientation tuning under range of overall illumination magnitudes

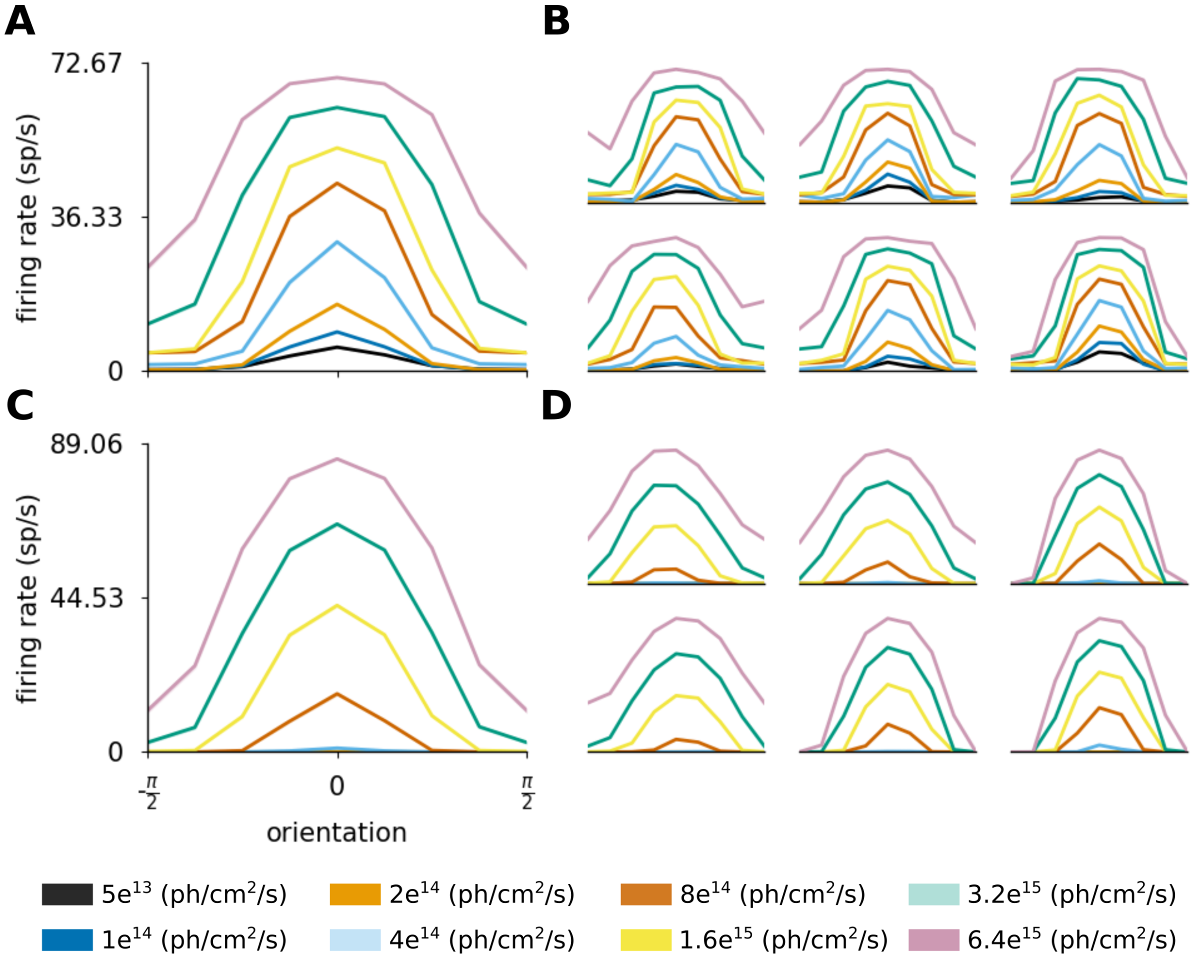

Figure S6: The impact of the diameter of individual light emitting elements on the induction of orientation tuning in V1. Each column corresponds to cortical optogenetic stimulation simulation with different size and pitch of light emitting elements. (A) The illumination intensity at the cell body as a function of the orientation preference of the given neuron. (B) The orientation tuning curves centered and averaged across all recorded neurons. (C) The scatter plot showing the orientation tuning width measured as HWHH at minimum and maximum contrast. (D) The histogram of tuning width measured as HWHH at maximum contrast. The black arrows on top mark the mean of the distribution. The red arrows mark the mean HWHH of the natural vision condition.

1.5 The impact of sharpness parameter of stimulation protocol on the OptoExcInh condition

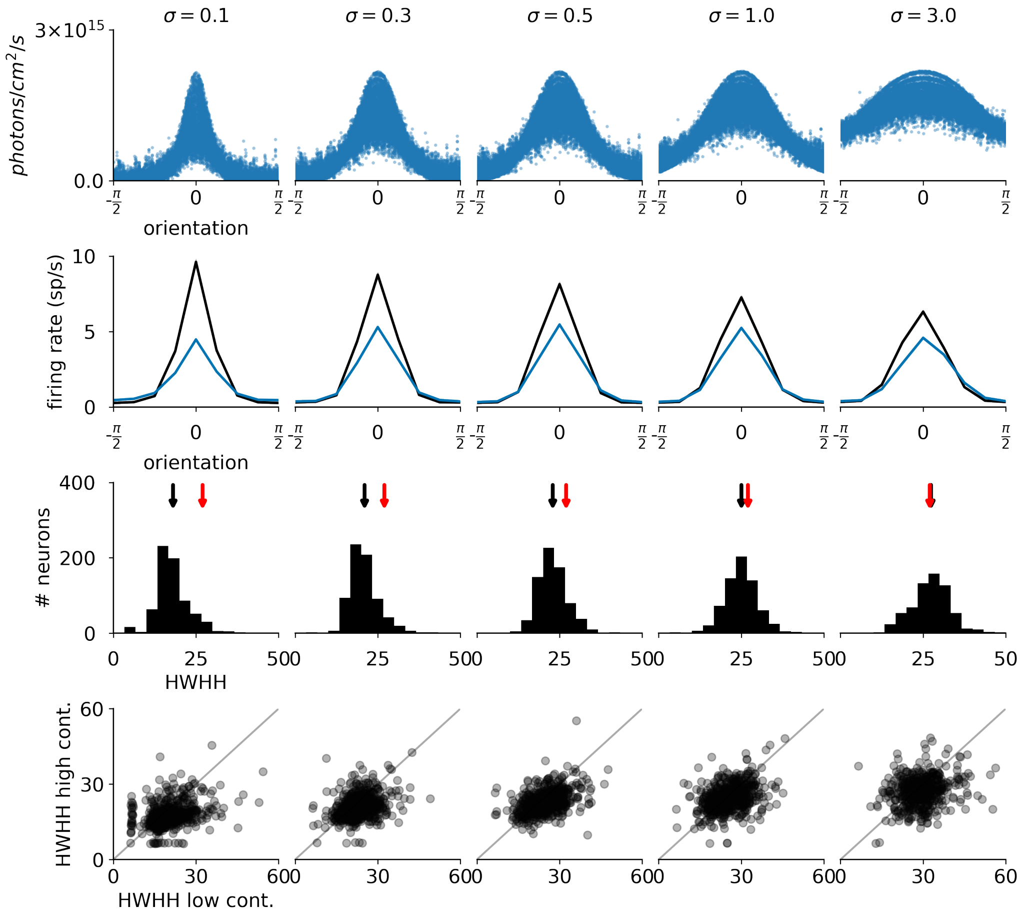

Figure S7: Width of orientation tuning as a function of the sharpness parameter of the optogenetic stimulation protocol in the OptoExcInh condition. Each column corresponds to cortical optogenetic stimulation simulation where different parameters of the stimulation orientation sharpness σ were used. (A) Illumination intensity at the cell body as a function of the orientation preference of the given neuron. (B) Mean of the orientation tuning curves across all recorded neurons (after realignment on their respective orientation preference). (C) The scatter plot showing the orientation tuning width measured as HWHH at low (abscissa) vs. high (ordinate) contrast. (D) The histogram of tuning width measured as HWHH at maximum contrast. The black arrows on top mark the mean of the distribution. The red arrows mark the mean HWHH of the visually-driven condition.

1.6 The impact of light emitting element diameter on the OptoExcInh condition

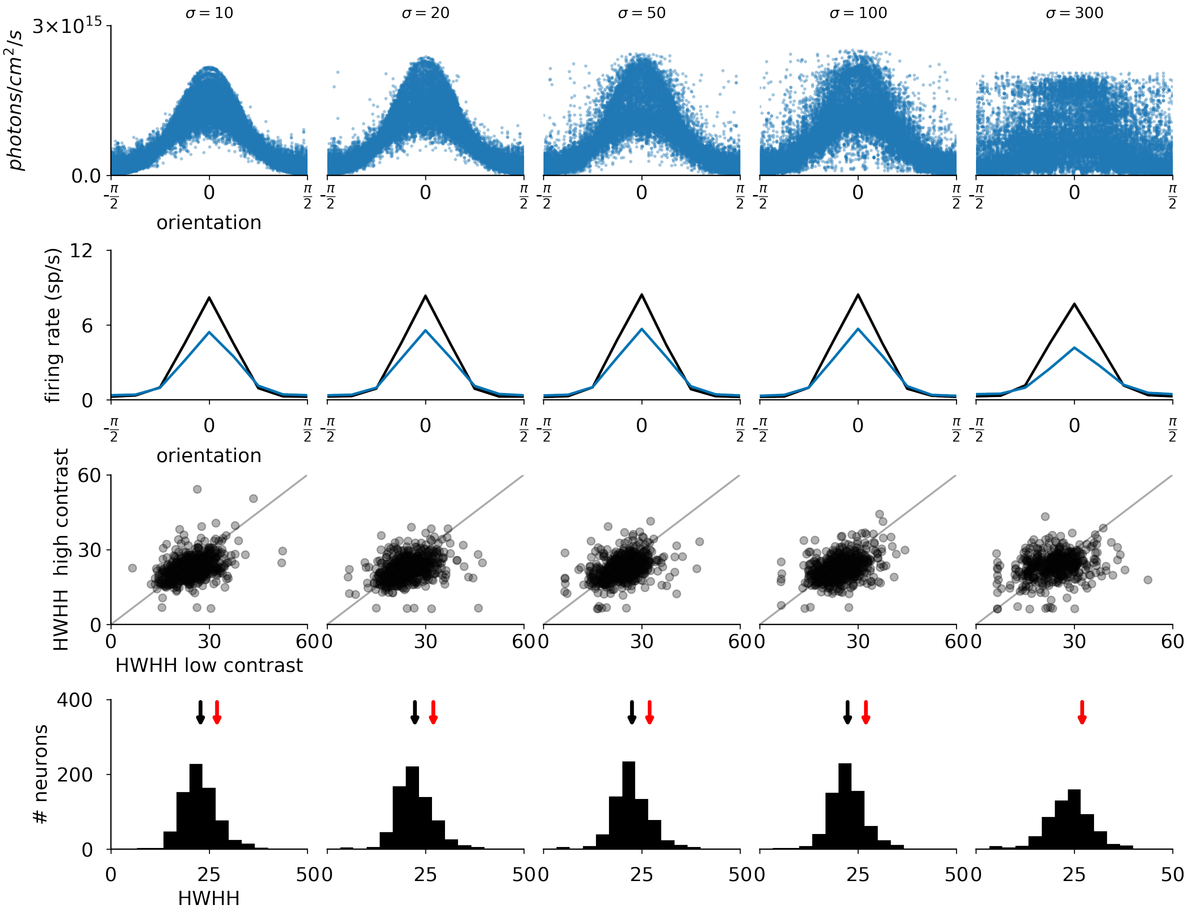

Figure S8: The impact of the diameter of individual light emitting elements on the induction of orientation tuning in V1. Each column corresponds to cortical optogenetic stimulation with different size and pitch of light emitting elements in the OptoExcInh condition. (A) The illumination intensity at the cell body as a function of the orientation preference of the given neuron. (B) The orientation tuning curves centered and averaged across all recorded neurons. (C) The scatter plot showing the orientation tuning width measured as HWHH at low (abscissa) vs. high (ordinate) contrast. (D) The histogram of tuning width measured as HWHH at maximum contrast. The black arrows on top mark the mean of the distribution. The red arrows mark the mean HWHH of the NatVis condition.

References

[1] Cossart, R., Aronov, D. & Yuste, R. Attractor dynamics of network UP states in the neocortex. *Nature* **423**, 283–288 (2003).

[2] Shu, Y., Hasenstaub, A. & McCormick, D. A. Turning on and off recurrent balanced cortical activity. *Nature* **423**, 288–293 (2003).

[3] Esposito, F., Mulert, C. & Goebel, R. Combined distributed source and single-trial EEG-fMRI modeling: Application to effortful decision making processes. *NeuroImage* **47**, 112–121 (2009).

[4] Lee, A. K., Manns, I. D., Sakmann, B. & Brecht, M. Whole-Cell Recordings in Freely Moving Rats. *Neuron* **51**, 399–407 (2006).

[5] Destexhe, A. & Rudolph-Lilith, M. *Neuronal noise*. Springer Series in Computational Neuroscience (Springer US, 2012). `arXiv:1011.1669v3`.

[6] Bringuier, V., Frégnac, Y., Baranyi, A., Debanne, D. & Shulz, D. E. Synaptic origin and stimulus dependency of neuronal oscillatory activity in the primary visual cortex of the cat. *Journal of Physiology* **500**, 751–774 (1997).

[7] Swadlow, H. A. Fast-spike Interneurons and Feedforward Inhibition in Awake Sensory Neocortex. *Cerebral Cortex* **13**, 25–32 (2003).

[8] Monier, C., Chavane, F., Baudot, P., Graham, L. J. & Frégnac, Y. Orientation and direction selectivity of synaptic inputs in visual cortical neurons: a diversity of combinations produces spike tuning. *Neuron* **37**, 663–80 (2003).

[9] Bardy, C., Huang, J. Y., Wang, C., FitzGibbon, T. & Dreher, B. 'Simplification' of responses of complex cells in cat striate cortex: Suppressive surrounds and 'feedback' inactivation. *Journal of Physiology* **574**, 731–750 (2006).

[10] Monier, C., Fournier, J. & Frégnac, Y. In vitro and in vivo measures of evoked excitatory and inhibitory conductance dynamics in sensory cortices. *Journal of Neuroscience Methods* **169**, 323–365 (2008).

[11] Hromadka, T., DeWeese, M. R. & Zador, A. M. Sparse Representation of Sounds in the Unanesthetized Auditory Cortex. *PLoS biology* **6**, 124–137 (2008).

[12] Buzsáki, G. & Mizuseki, K. The log-dynamic brain: how skewed distributions affect network operations. *Nature Reviews Neuroscience* **15**, 264–278 (2014). `NIHMS150003`.

[13] Nowak, L. G., Sanchez-Vives, M. V. & McCormick, D. A. Lack of orientation and direction selectivity in a subgroup of fast-spiking inhibitory interneurons: Cellular and synaptic mechanisms and comparison with other electrophysiological cell types. *Cerebral Cortex* **18**, 1058–1078 (2008). `NIHMS150003`.

[14] Cardin, J. A., Palmer, L. A. & Contreras, D. Stimulus feature selectivity in excitatory and inhibitory neurons in primary visual cortex. *Journal of Neuroscience* **27**, 10333–10344 (2007).

[15] Finn, I. M., Priebe, N. J. & Ferster, D. The emergence of contrast-invariant orientation tuning in simple cells of cat visual cortex. *Neuron* **54**, 137–52 (2007).
